# Defining novel plant polyamine oxidase subfamilies through molecular modeling and sequence analysis

**DOI:** 10.1186/s12862-019-1361-z

**Published:** 2019-01-21

**Authors:** Cesar Daniel Bordenave, Carolina Granados Mendoza, Juan Francisco Jiménez Bremont, Andrés Gárriz, Andrés Alberto Rodríguez

**Affiliations:** 1 Laboratorio de Fisiología de Estrés Abiótico en Plantas, Unidad de Biotecnología, INTECH - CONICET - UNSAM, Intendente Marino KM 8.2 - B7130IWA Chascomús, Buenos Aires, Argentina; 2 Departamento de Botánica, Instituto de Biología, Universidad Nacional Autónoma de México, Apartado Postal 70-367, Coyoacán, 04510 México City, Mexico; 3 División de Biología Molecular, Instituto Potosino de Investigación Científica y Tecnológica (IPICYT), San Luis Potosí, Mexico

**Keywords:** Evolution, Phylogeny, Polyamine oxidase, Polyamine catabolism, Protein structure, Homology modeling

## Abstract

**Background:**

The polyamine oxidases (PAOs) catabolize the oxidative deamination of the polyamines (PAs) spermine (Spm) and spermidine (Spd). Most of the phylogenetic studies performed to analyze the plant PAO family took into account only a limited number and/or taxonomic representation of plant PAOs sequences.

**Results:**

Here, we constructed a plant PAO protein sequence database and identified four subfamilies. Subfamily PAO back conversion 1 (PAObc1) was present on every lineage included in these analyses, suggesting that BC-type PAOs might play an important role in plants, despite its precise function is unknown. Subfamily PAObc2 was exclusively present in vascular plants, suggesting that t-Spm oxidase activity might play an important role in the development of the vascular system. The only terminal catabolism (TC) PAO subfamily (subfamily PAOtc) was lost in Superasterids but it was present in all other land plants. This indicated that the TC-type reactions are fundamental for land plants and that their function could being taken over by other enzymes in Superasterids. Subfamily PAObc3 was the result of a gene duplication event preceding Angiosperm diversification, followed by a gene extinction in Monocots. Differential conserved protein motifs were found for each subfamily of plant PAOs. The automatic assignment using these motifs was found to be comparable to the assignment by rough clustering performed on this work.

**Conclusions:**

The results presented in this work revealed that plant PAO family is bigger than previously conceived. Also, they delineate important background information for future specific structure-function and evolutionary investigations and lay a foundation for the deeper characterization of each plant PAO subfamily.

**Electronic supplementary material:**

The online version of this article (10.1186/s12862-019-1361-z) contains supplementary material, which is available to authorized users.

## Background

PAOs are amino oxidases involved in polyamine metabolism. This group of enzymes catalyzes the oxidation of free higher PAs such as Spm and Spd, and their acetylated derivatives at their secondary amino groups through two known reaction modes [1]. Thus, PAOs acting in the TC of PAs oxidize the carbon on the *endo*-side of the N_5_ of Spm or Spd producing 1,3-diaminopropane (DAP), H_2_O_2_, and the respective aldehydes [2]. In turn, PAOs functioning in the BC pathway oxidize the carbon on the *endo*-side of the N_5_ of Spm and Spd rendering Spd and putrescine (Put), respectively, as well as 3-aminopropanal and H_2_O_2_ [2]. Put, Spd and Spm are the most abundant free PAs in plants [3], and the oxidation of these amines have been associated with numerous events related to cell growth and development, biotic and abiotic stress responses [4–8].

Plant PAOs show a great heterogeneity in terms of reaction mode, substrates, products and subcellular localization [9–19]. However, the current knowledge about aspects of plant PAOs is limited to enzymes from a few model plant species like *Z. maize*, *A. thaliana*, *O. sativa*, *G. hirsutum* and *B. distachyon* [9–21]. On the other hand, whereas three PAO protein crystal structures are available at the PDB database, only one of them represents the plant kingdom: the maize apoplastic TC-type PAO1 (ZmPAO1) [22–25]. The other two PAO crystals structures belong to the *S. cerevisiae* BC-type PAO (FMS1) [26] and the *M. musculus* N1-acetyl PA oxidase (MmAPAO) [21, 27]. Although ZmPAO1 has been used as template to model the structure of mammalian PAOs [28], molecular-modeling structure analysis of plant PAOs are scarce [29, 30].

The first aim of the present work was to build a protein sequence database, through a domain architecture analysis strategy [31], in order to investigate the evolutionary relationships among plant PAOs including an ample taxonomic representation of the main angiosperm lineages. The second aim was to analyze the structural features and the conservation of the amino acids involved in the active site of plant PAOs. In order to do this, we performed an analysis of protein structures obtained by molecular modeling using the available crystal structures of PAOs. Our results showed that the plant PAO family is composed of at least four subfamilies with distinct evolutionary relationships, structural and functional features. In addition, their analysis allowed us to identify the amino acids potentially involved in the enzymatic mechanism.

## Results

### Sequence database construction through a domain architecture approach

The election of the remote-homology detection method is an important factor when searching for plant PAOs sequences, since the majority of the known members of this group show low sequence identity (Additional file 1 Table S1). In this trend, the Pfam database, a domain architecture HMM-based database [32], is probably the most appropriate source of sequences. Therefore, we searched the Pfam database for domains representing amino oxidase enzymes.

Domain architecture analysis revealed the presence of a single copy of the *Amino_oxidase* domain (PF01593) and the absence of other domains in any of the sequences analyzed (Additional file 1 Figure S1). After filtering the sequences, the database comprised 543 sequences from 46 angiosperm species (17 monocot and 29 eudicot species, and *Amborella*) and 124 sequences from early divergent green plants (the Chlorophytes *Chlorella variabilis*, *Micromonas pusilla* and *Chlamydomonas reinhardtii,* the Charophyta *Klebsormidium nitens*, the Bryophyta *Physcomitrella patens*, the Lycopodiophyta *Selaginella moellendorffii*, the Monilophyta *Azolla filiculoides* and the Gymnosperm *Araucaria cunninghamii*).

### Clustering of the sequence database

**Fig. 1:**
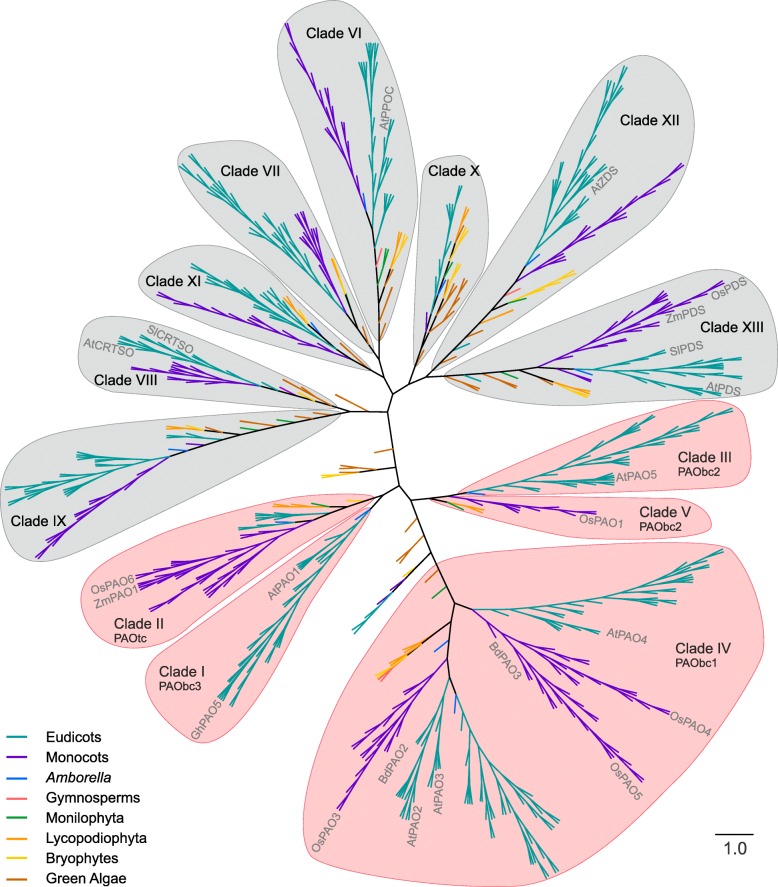
Rough clustering of the sequence database. A distance tree was obtained by rough clustering using the UPGMA method. The names of the well-documented plant PAOs and characterized enzymes as non PAO are indicated in grey within each clade. *A. thaliana* Protoporphyrinogen oxidase 1, Prolycopene isomerase, Zeta-carotene desaturase and Phytoene desaturase (AtPPOC, AtCRTSO, AtZDS and AtPDS); *S. moellendorffii* Prolycopene isomerase and Phytoene desaturase (SlCRTSO and SlPDS); *Z. mays* Phytoene desaturase (ZmPDS); *O. sativa* Phytoene desaturase (OsPDS). Grey colored clades correspond to non-PAO groups

Sequences were clustered using a distance method (UPGMA) and sequences from green plants species were classified into thirteen clades (Fig. 1; Additional file 1 Table S2). A new set of alignments within each clade showed that each of them comprehended a unique group of homologous sequences.

Clade I contained 31 sequences from eudicots species, including the sequences of *A. thaliana* PAO1 (AtPAO1) and *G. hirsutum* PAO5 (GhPAO5), both BC-type PAOs [10, 17]. Clade II was almost exclusively composed of genes from monocots species (34 sequences from monocots out of 37 total sequences), including the sequences of the apoplastic enzymes ZmPAO1 and *O. sativa* PAO6 (OsPAO6) [18, 22, 33]. Even though ZmPAO1 is a well-documented TC-type PAO [20], OsPAO6 reaction mode has not yet been determined. Clade III showed 34 sequences from eudicots species, including the sequence of *A. thaliana* PAO5 (AtPAO5), a cytoplasmic BC-type PAO [14]. Clade IV included 134 sequences, 81 from eudicots and 53 from monocots species. Among these sequences were found BC-type PAOs such as *A. thaliana* PAOs 2 to 4 (AtPAO2, AtPAO3, AtPAO4), *O. sativa* PAOs 3 to 5 (OsPAO3, OsPAO4, OsPAO5) and *B. distachyom* PAOs 2 and 3 (BdPAO2, and BdPAO3) [11, 13, 19, 34, 35]. Interestingly, AtPAO2, AtPAO3, AtPAO4, OsPAO3, OsPAO4 and OsPAO5 were reported as peroxisomal enzymes [11, 13, 35]. Clade V was composed of seven sequences from monocots species, including the cytoplasmic BC-type PAO *O. sativa* PAO1 (OsPAO1) [15]. Clades I to V did not included any sequences of proteins previously reported and characterized as non PAO enzymes. At last, Clades VI to XIII did not include any sequence belonging to enzymes with reported PAO activity, however some of these sequences were previously reported and characterized as non PAO enzymes (Fig. 1). Features of the well-documented PAOs within each clade were overviewed in Table 1.

**Table 1:** Known plant PAOs summary table

Name	Clade	UniproKB ID	Reaction mode	Substrate	Products	Subcellular Localization	Bibliography
AtPAO1	1	Q9FNA2	BC	Spm, N1-ac-Spm, Nor-Spm, t-Spm	Spd + H_2_O_2_	Cytoplasmic	[10, 34, 35]
GhPAO5	1	IDL7VGD5	BC	Spm	Spd + H_2_O_2_	–	[17]
ZmPAO1	2	O64411	TC	Spd, Spm	DAP + H_2_O_2_	Apoplastic	[22, 23, 33, 36]
OsPAO6	2	Q0J291	–	–	–	Apoplastic	[18]
OsPAO7	2	Q0J290	TC	Spd, Spm, N1-Ac-PAs	DAP + H_2_O_2_	Apoplastic	[16]
AtPAO5	3	Q9SU79	BC	t-Spm, Spd, Spm, N1-Ac-PAs	Spd/Put + H_2_O_2_	Cytoplasmic	[14]
AtPAO2	4	Q9SKX5	BC	Spd, Spm, N1-Ac-PAs, tSpm, Nor-Spm	Spd/Put + H_2_O_2_	Peroxisomal	[11, 34, 35]
AtPAO3	4	Q9LYT1	BC	Spd, Spm, N1-Ac-PAs, tSpm, Nor-Spm	Spd/Put + H_2_O_2_	Peroxisomal	[11, 34, 35]
AtPAO4	4	Q8H191	BC	Spd, Spm, tSpm	Spd/Put + H_2_O_2_	Peroxisomal	[11, 34, 35]
BdPAO2	4	I1J1Z5	BC	Spd, Spm, N1-Ac-PAs, tSpm	Spd/Put + H_2_O_2_	–	[19]
BdPAO3	4	I1J380	BC	Spm	Spd + H_2_O_2_	–	[19]
OsPAO3	4	Q7X809	BC	Spd, Spm, Nor-Spm	Spd/Put + H_2_O_2_	Peroxisomal	[13]
OsPAO4	4	Q7XR46	BC	Spd, Spm, Nor-Spm	Spd + H_2_O_2_	Peroxisomal	[13]
OsPAO5	4	Q0J954	BC	Spd, Spm, Nor-Spm	Spd + H_2_O_2_	Peroxisomal	[13]
OsPAO1	5	Q5NAI7	BC	tSpm, Spm, N1-Ac-Spm, Nor-Spm	Spd + H_2_O_2_	Cytoplasmic	[15]

Although there are differences in the subcellular localization between known PAOs of different clades (Table 1), the results of the prediction of subcellular localization were not consistent and therefore they were not included as a classification criteria (Additional file 1: Table S3).

### Protein structure homology-modeling of plant PAOs

The protein sequences of all clades were modeled with the three available PAO crystal structures. ZmPAO1 resulted in the best template for Clades I and II, whereas members of Clades III to V were best modeled with MmAPAO. In turn, low quality models were obtained with the sequences included in Clades VI to XIII. Therefore, these results suggested that only the clades I to V belong to plant PAO subfamilies.

**Fig. 2:**
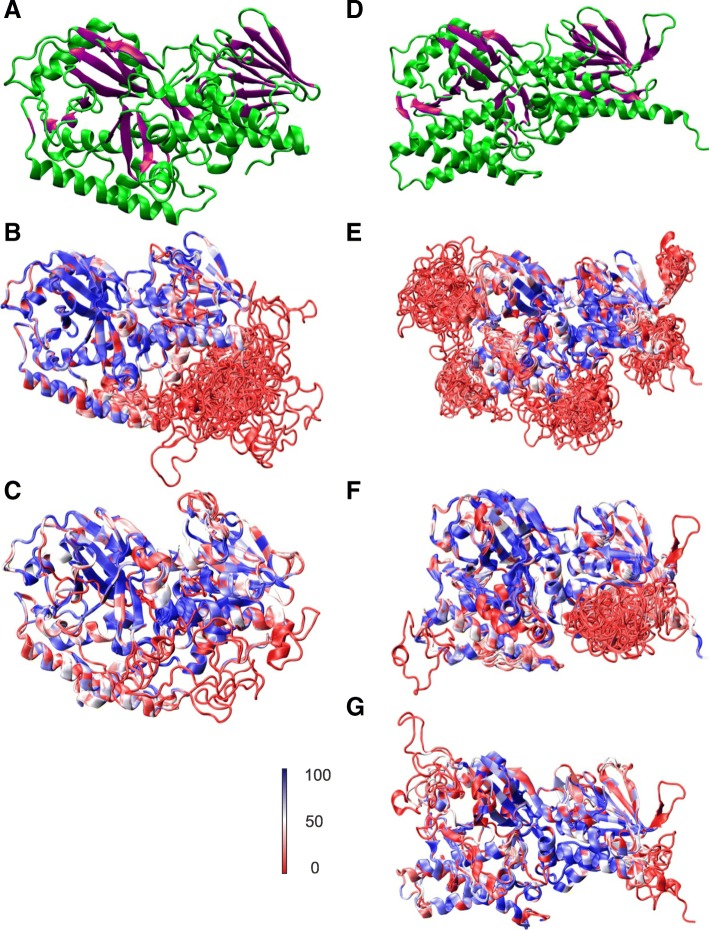
Protein structure of plant PAOs subfamilies. Protein structures were obtained by molecular modeling against the appropriate template. ZmPAO1 and MmAPAO templates showed on top colored by secondary structure, (**a**) and (**d**) respectively. Each picture shows an overlap of the structures within each group of sequences. The color represents percentage of identity. (**b**) Clade I. (**c**) Clade II. (**e**) Clade III. (**f**) Clade IV. (**g**) Clade V

Sequences of clades I to V were then compared to their best templates. Despite the variation in the inherent quality associated to the models, the structures obtained for each group showed a high consistence and a good percentage of identity in the core of the structures (Fig. 2). However, some of the groups showed portions of the sequences whose structure could not be determined.

The conservation of key amino acids at the active site was studied and compared among the clades (Table 2 and Additional file 1 Table S4). Most of the amino acids that constitute the active site were highly conserved within each group, and all the plant PAO subfamilies displayed the conservation of a lysine at the position of the residue Lys300 of ZmPAO1, which forms the catalytically essential structural motif Lys-H_2_O-FAD [25, 36]. In addition, Glu60 of ZmPAO1 is also present in all members of clade II, whereas those of clades III and IV exhibited a His in the same position, and clades I and V displayed the conservation of the non-polar side chain amino acids Ala and Gln, respectively. Interestingly, Glu60 of ZmPAO1 has been considered one of the most relevant amino acids in terms of interaction with the substrate, and it is substituted by His in MmAPAO, FMS1 and mammalian spermine oxidase (MmSMO) [24, 25, 36, 37].

**Table 2:** Plant PAOs active site analysis

ZmPAO1^a^	Tyr437	Phe401	Glu168	Tyr296	Glu60	Tyr167	Tyr163	Asn232	Lys300
MmAPAO^a^	Thr469	–	Val182	Tyr428	His59	Tyr194	Ser468	Glu179	Lys305
Clade I	Tyr	Tyr	Glu	Tyr	Ala	Phe	Ile	Asn	Lys
Clade II	Tyr	Phe/Tyr	Glu	Tyr	Glu	Tyr	Tyr/Phe	Asn	Lys
Clade III	Thr	–	Tyr	Tyr	His	Tyr	Ser	Gln	Lys
Clade IV	Ser	–	Met/Leu	Tyr	His	Glu	Gly	–	Lys
Clade V	Thr	–	Asp	Tyr	Gln	Tyr	Ser	Glu	Lys

^a^ZmPAO1 and MmAPAO are included as reference

The ZmPAO1 enzyme shows a Phe residue (Phe401) that is positioned parallel to a Tyr (Tyr437), both flanking the catalytic tunnel on opposite sides. These residues are thought to define a kind of *aromatic sandwich* around the substrate [37]. Our analysis showed that aromatic residues were conserved in these positions in clades I and II, but were absent in the other subfamilies (Table 2).

### Plant PAO subfamilies phylogenetic distribution

Plant PAO subfamilies showed a distinct phylogenetic distribution as a result of gene duplication and extinction events. Gymnosperms proteomes are still poorly documented, therefore no extinction event was hypothesize for these lineage.

**Fig. 3:**
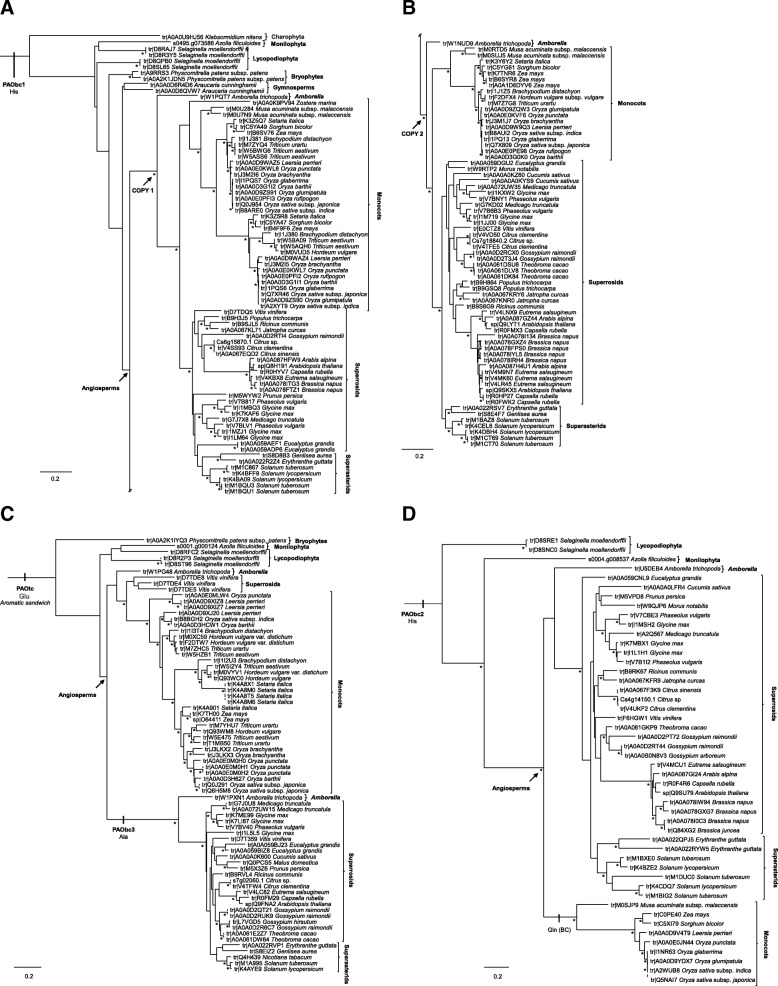
Phylogeny of plant PAOs subfamilies. The trees were constructed with maximum likelihood method. Nodes with Bootstrap support ≥85 are denoted by an asterisk. Aminoacid under the subfamily names indicates the residue present in the position corresponding to Glu60 of ZmPAO1. (**a**) Subfamily PAObc1. (**b**) Subfamilies PAOtc and PAObc3. (**c**) Subfamily PAObc2

**Fig. 4:**
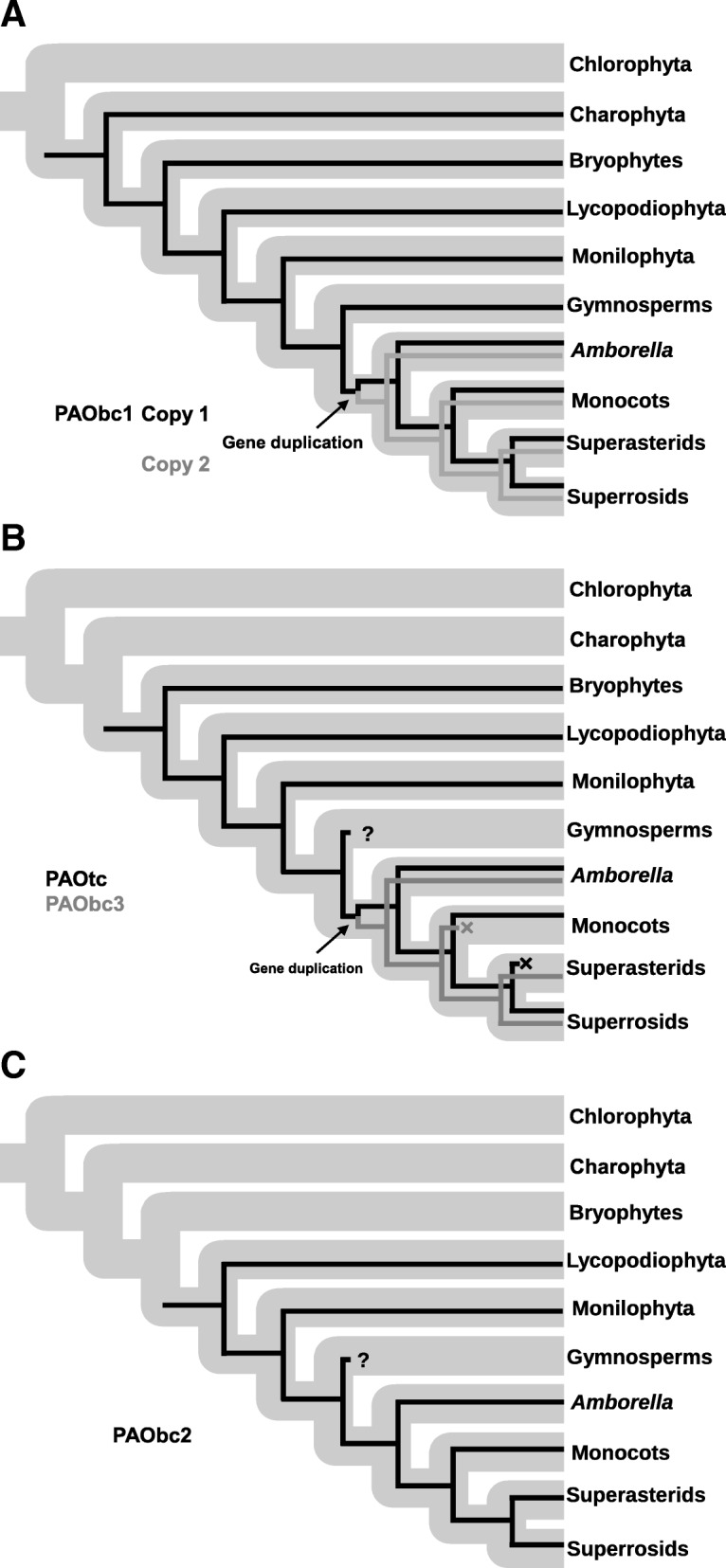
Evolution of plant PAO subfamilies. Grey thick branches correspond to the green plants species tree as recovered by Gitzendanner an collaborators [61]. The black and grey thin branches denotes the gene trees for each plant PAO subfamily. Gene extinction is denoted by an X, whereas? indicates uncertainty in the actual absence of the subfamilies in Gymnosperms. (**a**) Subfamily PAObc1. (**b**) Subfamily PAOtc and PAObc3. (**c**) Subfamily PAObc2

Our results indicate that clade IV conform a plant PAO subfamily, here after referred as PAObc1. This subfamily is present in all the main lineages of Streptophytes (including Gymnosperms), and that a gene duplication even (both copies with BS > 85) occurred along with the Angiosperms origin (Fig. 3a and b and Fig. 4a). A second subfamily, PAOtc (formerly referred as clade II), arose before the embryophytes diversification, subsequently having at least one gene extinction event in Superasterids (Fig. 3c and Fig 4b). Clade I, here after subfamily PAObc3, is the result of a gene duplication event preceding Angiosperm diversification (BS > 85), followed by a gene extinction in Monocots (Fig. 3c and Fig 4b. Subfamily PAObc2, comprehending clades III and V, is exclusively present in single copy in vascular plants (except Gymnosperms, but see above).

### Identification of motifs in plant PAOs subfamilies

Differential conserved protein motifs were found for each subfamily of plant PAOs (Table 3). A random subset of sequences from every group was scanned against the differential conserved motifs and the results were contrasted with the phylogenetic tree localization. All the sequences were assigned to the specific plant PAO subfamily using this approach (Table 4).

**Table 3:** Aminoacidic motifs for the identification on plant PAOs subfamilies

	Fitness	Hits (Seqs)	Motif
Subfamily 1	169,4	32 (32)	EFF[IM]YAH[DE][QR]RGY[FY][AT]FWQxM[DE]NA[HY]xGS[DN][IM]LVVT[LV]TN[DEG][EQ]Sx[HR][IV]E[ANS]Qx[DN]x[DE]T[LM]
Subfamily 2	134,0	37 (37)	T[DEK][IV]xV[LP]RWxS[DN][KR]F[FY]xG[ST][FY]SNxP[IV]GV[DNS][HR]x[EGQ][FHY][DN]x[IL][KR]APVGx(3)[FY]x[GT][EG][EH]x[ST][AEQS]
Subfamily 3	112,8	43 (43)	V[FV]E[AG]G[ADGNST]R[AIV]GGR[IV]x[ST]x[EQ]Fx[AGS]x(2)[IV]ExGATW[IV]xGx(1,2)Gx(1,2)P[ILV][HY]x[ILM][AS]x[DEQ]x(3)[FL]
Subfamily 4	125,1	136 (136)	LHG[AV][CGS]x[DE]N[PS][LV][AS]x(2)Ix(2)LxLx[IL]YxT[CGS]x[DG][DN]S[IV][IL][FY][DE]HDL[EK]Sx(2)[IL][FY][DN]x(2)Gx(2)[IV][EPS]

**Table 4:** Automatic subfamily assignment by aminoacidic motif search

ID	Organism	Assignation by phylogeny	Assignation by motif search
U5G093	*P. trichocarpa*	Subfamily 1	Subfamily 1
A0A078CR66	*B. napus*	Subfamily 1	Subfamily 1
M0XC59	*H. vulgare*	Subfamily 2	Subfamily 2
B8BGH2	*O. sativa*	Subfamily 2	Subfamily 2
R0F4R6	*C. rubella*	Subfamily 3	Subfamily 3
A0A087GI24	*A. alpina*	Subfamily 3	Subfamily 3
C5XI79	*S. bicolor*	Subfamily 3	Subfamily 3
A0A0D9V4T9	*L. perrieri*	Subfamily 3	Subfamily 3
R0FMX3	*C. rubella*	Subfamily 4	Subfamily 4
I1J381	*B. distachyon*	Subfamily 4	Subfamily 4
Lus10020726	*L. usitatissimum*	Not Included	No Hit
Lus10005021	*L. usitatissimum*	Not Included	Subfamily 4
Lus10039599	*L. usitatissimum*	Not Included	No Hit
Lus10019725	*L. usitatissimum*	Not Included	No Hit
Gh_A05G0221	*G. hirsutum*	Not Included	Subfamily 3
Gh_A07G0104	*G. hirsutum*	Not Included	Subfamily 3
Gh_A08G0331	*G. hirsutum*	Not Included	Subfamily 4
Gh_A08G0507	*G. hirsutum*	Not Included	Subfamily 1
Gh_A12G2582	*G. hirsutum*	Not Included	Subfamily 4
Gh_D05G0300	*G. hirsutum*	Not Included	Subfamily 3
Gh_D07G2378	*G. hirsutum*	Not Included	Subfamily 3
Gh_D08G0428	*G. hirsutum*	Not Included	Subfamily 4
Q0J290	*O. sativa*	Not Included	Subfamily 2
Cs4g14150.1	*C. cinnensis*	Not Included	Subfamily 3

Recently, some authors reported protein sequences from cotton and flax, characterized as plant PAOs in silico [17, 38]. These sequences were scanned against the differential conserved motifs being most of them (13 out of 16 sequences) assigned to a unique plant PAO subfamily (Table 4). This procedure was also carried out with the sequences of two well-documented plant PAOs that had not been considered in our previous analysis. *O. sativa* PAO7 (OsPAO7) protein [16], which was not included as it sequence did not passed the selection criteria (it had less than five amino acids before the start of the *Amino_oxidase* domain) and *C. cinencis* PAO4 (CsPAO4) [39], that was not included as it sequence was not present in the Pfam or UniProtKb databases. These sequences were scanned against the differential conserved motifs, and they were assigned to the plant PAO subfamily 2 and 3, respectively (Table 4).

## Discussion

A critical task when constructing a protein database suitable for phylogenetic analysis is the functional and structural characterization of new proteins. This is often inferred on the basis of the sequence similarities to proteins with known structure or function. However, remote-homologues [40] can be difficult to detect when distantly related proteins are analyzed using homologues-assigning methods based on pairwise procedures [41]. In this regard, Hidden Markov model (HMM) based methods have been applied to detect distantly related proteins with better results [40, 41]. In the first part of this work we built a protein sequence database of plant PAOs through a domain architecture analysis strategy using the Pfam database, a domain architecture HMM-based database [32]. Our sequence analysis revealed that all the proteins with reported PAO activity presented a single copy of the *amino_oxidase* domain without the presence of any other additional domain (Additional file 1 Figure S1). Even though proteins with single domains are unusual [42], this feature was useful to perform the sequence search and to establish filter criteria. Moreover, some clades included sequences of proteins previously reported and characterized as non PAO enzymes indicating a possible phylogenetic relation between these enzymes and revealing the versatility of the PAO domain architecture. The sequences in the final database shared the same domain architecture, but they did not define a unique group of homologous sequences. This was in line with the observation that the percentages of identity among some of the well-documented plant PAOs were lower than expected for homologous sequences (Additional file 1 Table S1) [43]. However, the clades detected in this work were constituted by unique groups of homologous sequences. Even though the distance trees constructed with the sequence database showed a similar topology as previous plant PAO phylogenetic studies [13, 15, 16, 34, 44, 45], it is noteworthy that we used a larger sequence database. Therefore, it was possible to build groups that included higher number of sequences and species (an average of 40 sequences and 26 species per clade) and to detect a higher number of clades than previous reports. This suggested that the plant PAO family is bigger than previously conceived. Although we only considered five of the thirteen clades as part of plant PAO subfamilies, we cannot rule out that other clades constitute subfamilies of these plant enzymes with a structure that cannot be modeled with any of the currently available PAO crystal structures.

**Fig. 5:**
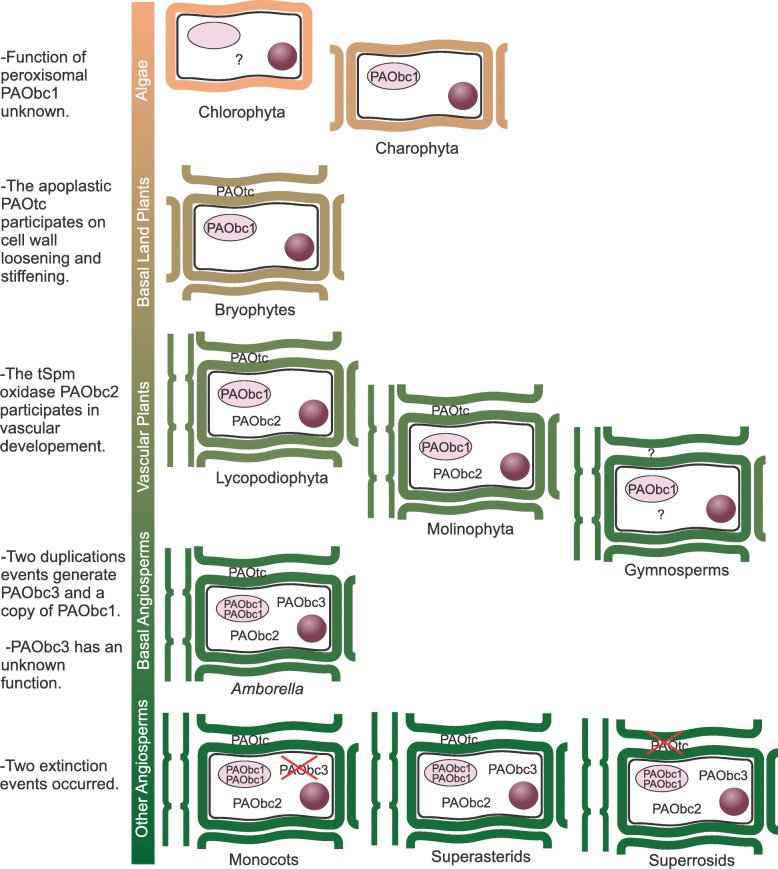
Hypothetical scenario for diversification of PAO in plant evolution. Distribution and subcellular localization of plant PAOs subfamilies are schematized for each plant lineage. On the left the mayor events on plant PAO evolution are mentioned. Cell walls are represented in color, nucleus are represented by a solid sphere, peroxisomes are represented by an ellipse and vessels are represented by the vertical structure in the lefts of the cells of vascular plants. Red crosses indicates extinction events

We also investigated the evolutionary relationships among plant PAOs. Even though several phylogenetic studies of plant PAOs have been performed [13, 15, 16, 19, 34, 38, 44–47], most of them included only a limited number and/or taxonomic representation of plant PAOs sequences hindering the elucidation of the evolution of this protein family in plants. For this reason, we decided not only to enrich the database in terms of number of protein sequences and structures, but also to increase the taxonomic representation of the main green plant lineages. The breadth of our taxon sampling allowed us to determine the phylogenetic distribution of each plant PAO subfamilies. No plant PAO subfamily was identified for Chlorophyta, however this is most probably due to the ample sequence divergence of this lineage compared to the other green plant lineages. The low gymnosperm sequence count in the database (only four) question the correct representation of Gymnosperm sequences in the database and suggest us to be cautious with the interpretation of the absence of several plant PAO subfamilies in this group. Therefore, as stated before, no extinction event was hypothesized for this plant group. The plant PAObc1 subfamily is ubiquitous in almost all green plants and was most probably present since the ancestral Streptophytes (Fig. 5). The peroxisomal subcellular location reported for some PAObc1 sequences is in agreement with the presence on this subfamily in Charophyta, since they present peroxisomes that are more similar to those of land plants compared to other green algae [48]. The apoplastic subfamily PAOtc is involved on cell wall loosening and stiffening during plant development [5, 7]. A different enzyme or group of enzymes must be replacing this function in Superasterids, like copper-containing amine oxidases (a hypothesis that has been already discussed) [49, 50]. The PAObc2 is known to participate during vascular development and this is in agreement with the presence of this subfamily exclusively in vascular plants (Fig. 5). The gene duplication events that gave rise of both copies of PAObc1 and PAObc3 identified since the early diverged angiosperm *A. trichopoda* suggest that the newly acquired plant PAOs formed part of the new gene content that first appeared in the ancestral angiosperms, possibly as a result of the Zeta (seed plants) and/or Epsilon (angiosperms) genome duplications [51, 52].

To strengthen our study, we also analyzed the structural features and the conservation of the amino acids involved in the active site of plant PAOs. Even though it is plausible that proteins with similar structures share similar functions, the protein structure homology-modeling showed us a discrepancy for the subfamily 1 with regard to this statement. Thus, the best template for this group was the TC-type PAO ZmPAO1. However, all of their well-documented members were characterized as BC-type enzymes (Table 1) [10, 17]. The remaining subfamilies showed an agreement between the reaction mode of its members and the reaction mode of its structure modeling templates. Many reports associated the PAO reaction modes with the presence of particular residues in their active site or in their catalytic tunnels. The TC reaction mode has been related to the presence of Glu in the active site and an *aromatic sandwich* in the catalytic tunnel [36, 37]. In this trend, Glu60 has been largely accepted as one of the more relevant residues for catalysis [36, 37], in terms of interaction with the substrate or its accommodation within the active site of ZmPAO1, as it forms a hydrogen bound with the N_5_ atom of the substrate [44]. The residue in this position in the active site of the BC-Type PAOs was also suspected to be important for the catalysis, but in this case it is substituted by His [26, 28, 39, 47]. In line with this, in the ZmPAO1 active site the residues that compose the *aromatic sandwich* around the substrate have been proposed to be important to define the reaction mode of the enzyme [37]. Glu and the *aromatic sandwich* were presented in the subfamily PAOtc, in its orthologe in *Amborella* and in all the sequences from early divergent green plants related to this clade, in agreement with the reaction mode of their members as well as the template for homology-modeling of this group, ZmPAO1. Moreover, these features were absent in subfamilies PAObc1 and PAObc2, suggesting that the members of these groups are BC-type enzymes. On the other hand, subfamily PAObc3 showed the conservation of the residues that conforms the *aromatic sandwich*, but Ala was conserved in the position corresponding to Glu60. Also its orthologe in *Amborella* presented the *aromatic sandwich* and a basic residue in the position corresponding to Glu60. These results suggested that the *aromatic sandwich* is a structural feature that is only present in the phylogenetically related subfamilies PAOtc and PAObc3 and more likely unrelated with the reaction mode of the enzyme. The presence of Glu to interact with the N_5_ of the substrate might be a critical factor determining the accommodation of the substrate to be oxidized on the *endo* side of the N_5_. Moreover, our results suggested that the presence of either a basic or an uncharged residue might lead to a BC-type PAO reaction mode. In this regard, Tavladoraki et al. (2011) carried out site-directed mutagenesis experiments on the His that occupies this position in the MmSMO, and they reported that its substitution by Glu leads to enzyme inactivation [28]. It would be of great interest to perform a site-directed mutagenesis experiment on a member of the subfamily PAObc3, such as AtPAO1 or GhPAO5, as they are more structurally similar to ZmPAO1, to unravel the function of this key residue.

When the structural, and catalytic features and the phylogenetic distribution of the plant PAO subfamilies were analyzed together, the following conclusions could be drawn:

a) Subfamily PAObc1 was present on every lineage included in these analyses (Fig. 5) suggesting that BC-type PAOs might play an important role in plants, despite its precise function is unknown.

b) Subfamily PAObc2 was exclusively present in vascular plants included in these analyses (Fig. 5) suggesting that t-Spm oxidase activity might play an important role in the development of the vascular system.

c) The only TC-type PAO subfamily (subfamily PAOtc) was lost in Superasterids but it is present in all other land plants (Fig. 5). This indicated that the TC-type reactions are fundamental for land plants and that their function could being taken over by other enzymes in Superasterids, a hypothesis that was already suggested in previous reports [49, 50].

As we stated at the introduction of this work, the plant PAO family showed heterogeneity in terms of reaction mode, substrate specificity, reaction products, subcellular localization and structural features. Therefore, we made an effort to enrich the sequence database, grouping and characterizing the sequences and defining plant PAO subfamilies in order to obtain a more homogeneous an accurate classification of this enzyme family. The plant PAO subfamilies proposed here revealed that this protein family is conformed, at least in part, by homogeneous groups in terms of reaction mode and structural features.

The assessment of the correct evolutionary relationship between proteins and the assignment of an individual sequence to a functional or evolutionary group requires rigorous and time-consuming phylogenetic analyses and the use of differential conserved protein motifs could be an alternative approach to reach this goal. The automatic assignment using differential conserved motifs for each plant PAO subfamily was found to be comparable to the assignment by rough clustering and phylogenetic analysis performed in this work (Table 4). Furthermore, OsPAO7 was assigned to subfamily PAOtc (Table 4). This was consistent with the overall characteristics of this subfamily, as OsPAO7 is an apoplastic TC-type PAO (Table 1) [17]. On the contrary, CsPAO4 was not assigned to subfamily PAOtc as expected, given that it has been characterized as a TC-type PAO. Instead, it was located in subfamily PAObc2 (Table 4), which is in agreement with the sequence similarity of the members of this subfamily and with the lack of introns in the gene sequence, a particular feature shared with AtPAO5 [14, 45]. A further analysis revealed that CsPAO4 can be better modeled using MmAPAO as template, and the model obtained revealed the absence of the *aromatic sandwich* and the presence of His instead of Glu in the active site (Additional file 1: Table S5). Therefore, these motifs might be a useful tool for the identification *from scratch* of new plant PAOs as long as in the future the plant PAO subfamilies proposed in this work proves to be an accurate classification.

## Conclusion

The results presented in this work reveal that the plant PAO family is bigger than previously conceived and provides new information on sets of candidate plant PAO sequences offering a potential starting point for further experimental verifications. Besides, the models obtained through the structure modeling analysis revealed that the residue interacting with the N_5_ of the substrate PA might be one of the factors determining the reaction mode of the enzyme. Future additions to the structural and enzymatic properties of plant PAOs from different subfamilies may provide the necessary information needed to further characterize these groups. As an overall, this work delineates important background information for future specific structure-function and evolutionary investigations and lay a foundation for the in depth characterization of each plant PAO subfamily.

## Methods

### Domain architecture analysis

Domain architecture of all the currently well-documented PAO sequences was performed. ZmPAO1 (UniprotKB ID O64411), FMS1 (P50264), MmAPAO (Q8C0L6), AtPAO1 to 5 (Q9FNA2, Q9SKX5, Q9LYT1, Q8H191 and Q9SU79), OsPAO1 (Q5NAI7), OsPAO3 to 5 (Q7X809, Q7XR46 and Q0J954), OsPAO7 (Q0J290), BdPAO 2 and 3 (I1J1Z5 and I1J380), GhPAO5 (IDL7VGD5), SynPAO (C*yanobacterium synechocystis* PAO; Q6ZEN7) and BjPAO (*Braquiostoma japonicum* PAO; A0A059VBM4) were chosen since their kinetic properties, substrate specificity and reaction mode were well documented [9–21, 47, 53]. The protein sequences were scanned against the Pfam domain database using hmmscan software from the HMMER web server (http://www.hmmer.org/). Complete sequences were analyzed not excluding regions that that were not assigned to any domains.

### Data collection and database construction

An amino acidic sequence database based on the peptides domain architecture was constructed. In this regard, peptides sequences with a single *Amino_oxidase* domain (PF01593) and no other domains were selected from the PFAM database*.* Protein sequences with other domain architectures containing the *Amino_oxidase* domain and other extra domains are known to have functions different from PAO, and no PAOs have been reported containing other extra domains (i.e. plant lysine histone demethilases posses an extra SWIRM domain) [54, 55]. Sequences from angiosperms and other early divergent green plant species were retrieved and filtered with the following selection criteria based on the domain architecture analysis:

1. Less or equal to 50 amino acids missing on any side of the *Amino_oxidase* domain.
2. No s gap with less or equal than 150 amino acids long.
3. At least five amino acids before the start and five amino acids after the end of the *Amino_oxidase* domain (to avoid truncated sequences).
4. No more than 700 amino acids in length (to exclude proteins with additional domains that could not been currently identified by the Pfam software).

With the purpose of improving the taxonomic representation, the genome of the recently sequenced fern *Azolla filiculoides* was searched for protein sequences that matched the PAO domain architecture using the stand alone version of the HMMR software. Sequences that passed the selection criteria were added to the database.

### Global alignment and rough clustering

Sequences were aligned using the MAFFT online service [56], with a gap opening penalty of five. The resulting alignment was then manually adjusted and ambiguously aligned flanking regions were trimmed before subsequent clustering analysis.

For the rough clustering, a distance method (UPGMA) was used to construct a distance tree. Nodes separated by accumulative branch length less than 0.3 were considered to belong to the same cluster.

### Search for sequences annotated as a different enzyme other than PAOs

Sequences within the database were used to perform a search in UniprotKB. IDs with a annotated status of “Reviewed” were selected to perform a manual search of the bibliography. Proteins other than PAOs whose activity was determined were considered as a different enzyme.

### Prediction of subcellular localization

In order to predict subcellular localization of sequences within clades I to V, sequences were analyzed with a set of software using the standard configuration for plant protein sequences: SignalP 4.1 (cbs.dtu.dk/services/SignalP/), WoLF PSORT (wolfpsort.hgc.jp), LOCALIZER (localizer.csiro.au/), slplocal2 (sunflower.kuicr.kyoto-u.ac.jp/~smatsuda/slplocal.html), DeepLoc1,0 (cbs.dtu.dk/services/DeepLoc/), PredSL (aias.biol.uoa.gr/PredSL/), TargetP1.1 (cbs.dtu.dk/services/TargetP/) and Pprowler (bioinf.scmb.uq.edu.au:8080/pprowler_webapp_1–2/).

### Protein structure homology-modeling of plant PAOs

Three new alignments were made for each individual cluster including in each one the sequences of one of the three PAOs with known structure. Then, each sequence from the individual clusters was modeled using the ZmPAO1 (pdb code 3KU9), FMS1 (pdb code 1XPQ) and MmAPAO (pdb code 5MBX) structures as templates. For this, the *alignment mode* module from the SWISS-MODEL server was used (https://swissmodel.expasy.org/), with the corresponding alignment. Model quality parameters and *.pdb* files were retrieved. Although models with absolute QMEAN Z-score > 4 are considered to be of low quality [57] we decided to use a more lax criteria as the template for some models was taxonomically distant from the sequence to model. Models with more than 4.5 of absolute QMEAN Z-Score or less than 0.6 of GMQE were considered to be of low quality. Clusters whose sequences could not be modeled with any of the three templates (i.e. models obtained for every sequence were of low quality) were considered not belonging to plant PAO subfamilies.

### Phylogenetic analyses

PAO sequences from the green plant lineages Charophytas, Bryophytes, Lycopodiophyta, Monilophyta, and Gymnosperms, as well as respective sequences from *A. trichopoda*, the single living representative from the sister lineage to all other angiosperms, were incorporated into the dataset to increase the breadth of our taxonomic sampling within angiosperms. These sequences were retrieved and filtered from Pfam with the same criteria used on the construction of the database. A second round of alignments was performed with MAFFT online service, now with a gap opening penalty of three. The resulting alignments were then manually adjusted and ambiguously aligned regions were trimmed before subsequent analyses. The best fitting model of substitution was selected with PartitionFinder 2 [58]. Maximum Likelihood was used for phylogenetic reconstructions with the program RAxML HPC2 version 8.2.9 [59], applying the “rapid Bootstrap and search for best-scoring ML tree” algorithm.

### Structure analysis and active site amino acidic profiles

A multi-sequence structural analysis was carried out using the models obtained with the most suitable template for each subfamily. For this, the models obtained with the template that more frequently prompted the higher modeling quality parameters were selected for each subfamily and analyzed using the Multiseq module of the VMD software [60], applying the Stamp Structure Alignment tool.

The individual residues and its proposed equivalents were selected for analyzing based on previously published works [21, 24, 25, 36, 37].

### Differential conserved motifs identification

Motif search for automatic classification of plant PAOs were performed by using PRATT tool from ExPASy Bioinformatic Resource Portal (expasy.org/PRATT) using the aligned sequences for each plant PAO subfamily. Sequence scanning against motifs was carried out using the Scanprosite program (expasy.org/tools/scanprosite). Sequences of recent reports of plant PAOs not included on this work were used to test the automatic classification of plant PAOs by aminoacidic motif search.

## Additional file

Additional file 1: **Figure S1.** Domain Architecture of well-documented plant PAOs. **Table S1.** Percentages of identity between some of the well-documented plant PAOs. **Table S2.** Model quality parameters of models. **Table S3.** Prediction of subcellular localization. **Table S4.** Plant PAOs active site analysis. **Table S5.** CsPAO4 model quality and active site analysis. (PDF 12183 kb)

## Abbreviations

AtPAO1–5: *Arabidopsis thaliana* PAO 1–5
BC: back conversion
BdPAO2 and BdPAO3: *Brachypodium distachyon* PAO 2 and PAO 3
CsPAO4: *Citrus sinensis* PAO 4
DAP: 1,3-diaminopropane
FMS1: *Saccharomyces cerevisiae* PAO
GhPAO5: *Gossypium hirsutum* PAO 5
HMM: Hidden markov model
MmAPAO: *Mus musculus* N1-acetyl PA oxidase
MmSMO: *M. musculus* spermine oxidase
*Oryza sativa* PAO 1–7: OsPAO1–7
PAOs: polyamine oxidases
PAs: polyamines
Put: putrescine
Spd: spermidine
Spm: spermine
TC: terminal catabolism
ZmPAO1: *Zea mays* PAO 1
