# Supplementary material for: Defining novel plant polyamine oxidase subfamilies through molecular modeling and sequence analysis
Source: BMC Evol Biol. 2019 Jan 21;19:28. doi: 10.1186/s12862-019-1361-z (PMC6341606; doi:10.1186/s12862-019-1361-z)
Supplement: Supplementary file 1 — Figure S1. Domain Architecture of well-documented plant PAOs. Table S1. Percentages of identity between some of the well-documented plant PAOs. Table S2. Model quality parameters of models. Table S3. Prediction of subcellular localization. Table S4. Plant PAOs active site analysis. Table S5. CsPAO4 model quality and active site analysis. (PDF 12183 kb) [file 12862_2019_1361_MOESM1_ESM.pdf]

Figure S1

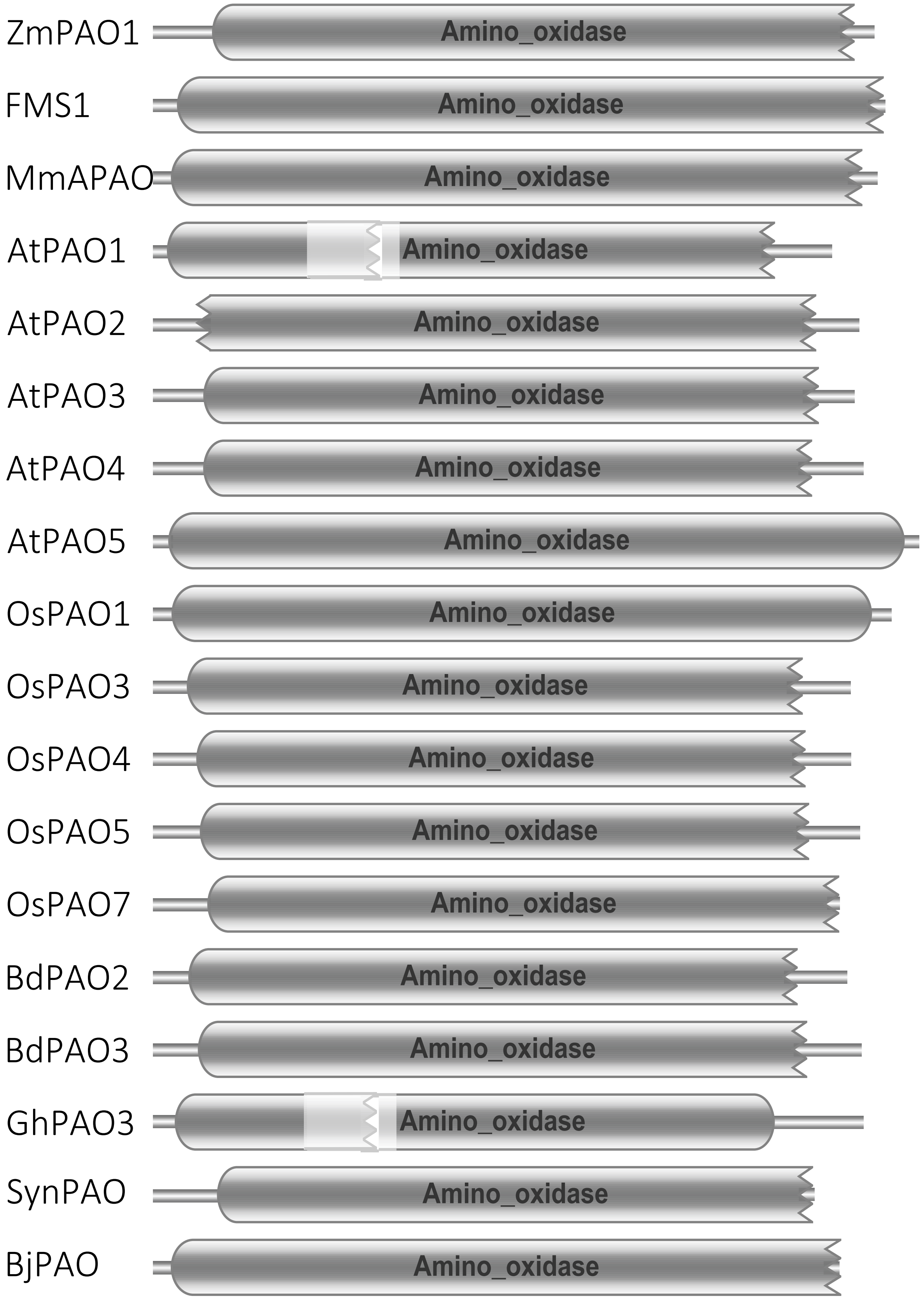

Figure S1: Domain Architecture of well characterized PAOs. Schemes of complete sequence are displayed. ZmPAO1 (*Z. mays* PAO1; UniprotKB ID O64411); FMS1 (*S. cereviceae* PAO; P50264); MmAPAO (*M. musculus* APAO; Q8C0L6); AtPAO1-5 (*A. thaliana* PAO1-5; Q9FNA2, Q9SKX5, Q9LYT1, Q8H191 and Q9SU79); OsPAO1 (*O. sativa* PAO1; Q5NAI7); OsPAO3-5 (*O. sativa* PAO3-5, Q7X809, Q7XR46 and Q0J954); OsPAO7 (*O. sativa* PAO7; Q0J290); BdPAO2-3 (*B. distachyon* PAO2 and PAO3; I1J1Z5 and I1J380); GhPAO5 (*G. hirsutum* PAO5; IDL7VGD5); SynPAO (*C. synechocystis* PAO; Q6ZEN7); BjPAO (*B. japonicum* PAO; A0A059VBM4).

Table S1

	ZmPAO1	AtPAO1	AtPAO2	AtPAO3	AtPAO4	AtPAO5	OsPAO1	OsPAO3	OsPAO4	OsPAO5	OsPAO7	BdPAO2	BdPAO3	GhPAO5
ZmPAO1	100													
atPAO1	48	100												
atPAO2	30	27	100											
atPAO3	30	28	85	100										
atPAO4	31	28	61	62	100									
atPAO5	24	23	24	24	25	100								
OsPAO1	45	23	27	26	29	42	100							
OsPAO3	30	28	73	72	61	25	27	100						
OsPAO4	30	28	63	63	60	27	29	60	100					
OsPAO5	29	26	65	62	63	27	25	61	76	100				
OsPAO7	82	47	33	29	29	23	24	29	29	28	100			
BdPAO2	29	26	71	73	59	24	26	95	61	62	29	100		
BdPAO3	28	26	63	62	60	27	28	62	84	74	27	61	100	
GhPAO5	48	80	29	28	30	41	23	28	27	27	48	27	27	100

Table S1: percentages of identity between some of the well documented plant PAOs. ZmPAO1 (Zea mays PAO1; UniprotKB ID O64411), atPAO1-5 (Arabidopsis thaliana PAO1-5; Q9FNA2, Q9SKX5, Q9LYT1, Q8H191 and Q9SU79); OsPAO1 (Oryza sativa PAO1; Q5NAI7); OsPAO3-5 (Oryza sativa PAO3-5, Q7X809, Q7XR46 and Q0J954); OsPAO7 (Oryza sativa PAO7; Q0J290); BdPAO2-3 (Brachypodium distachyon PAO2 and PAO3; I1J1Z5 and I1J380); GhPAO5 (Gossipium hirsutum PAO5; IDL7VGD5).

Table S2

Clade 1	ZmPAO1		FMS1		MmAPAO	
	GMQE	QMEAN	GMQE	QMEAN	GMQE	QMEAN
A0A022RVP1	0,69	-1,71	0,55	-5	0,58	-4,3
A0A059BIZ8	0,68	-0,42	0,53	-6,06	0,48	-4,85
A0A059BJ23	0,73	-0,9	0,58	-4,61	0,52	-4,91
A0A061DW64	0,68	-0,94	0,53	-5,76	0,57	-3,56
A0A061E2Z7	0,68	-0,92	0,53	-5,72	0,57	-3,4
A0A072UW15	0,71	-1,3	0,55	-5,21	0,6	-3,59
A0A078C0U9	0,69	-0,85	0,56	-4,49	0,52	-4,49
A0A078CR66	0,69	-1,17	0,56	-4,44	0,57	-3,19
A0A0A0K600	0,68	-0,8	0,53	-5,14	0,52	-5,22
A0A0D2QT21	0,68	-1,52	0,52	-6,01	0,56	-4,13
A0A0D2R6C7	0,68	-1,25	0,53	-5,58	0,57	-3,4
A0A0D2RUK9	0,68	-1,58	0,54	-5,91	0,57	-3,89
B9GFC0	0,67	-1,29	0,52	-4,85	0,56	-3,37
B9RVL4	0,68	-0,83	0,5	-7,59	0,56	-4,22
D7T359	0,67	-1,72	0,52	-6,42	0,56	-3,39
G7J0U8	0,66	-1,49	0,52	-6,3	0,56	-3,59
I1L5L5	0,72	-1,31	0,56	-6,13	0,6	-3,23
K4AYE9	0,66	-1,94	0,53	-5,92	0,55	-4,68
K7LI87	0,68	-1,37	0,57	-2,59	0,57	-2,59
K7ME99	0,67	-1,54	0,53	-5,17	0,5	-4,5
L7VGD5	0,67	-1,42	0,53	-5,6	0,56	-3,94
M1A995	0,67	-1,51	0,53	-6,21	0,53	-4,4
M5X3Z6	0,66	-0,89	0,52	-5,22	0,53	-2,56
Q9FNA2	0,71	-0,95	0,55	-5,19	0,57	-3,82
Q4H439	0,65	-1,94	0,53	-4,29	0,55	-4,38
R0FM29	0,53	-1,88	0,41	-5,43	0,45	-3,04
S8EIZ2	0,74	-0,85	0,59	-5,01	0,63	-3,5
U5G093	0,67	-1,09	0,52	-6,49	0,55	-4,4
V4LC82	0,7	-1,51	0,55	-5,48	0,58	-4,23
V4TFW4	0,67	-1,34	0,53	-5,41	0,51	-4,45
V7BV40	0,66	-1,29	0,51	-5,98	0,55	-2,99

Clade 2	ZmPAO1		FMS1		MmAPAO	
	GMQE	QMEAN	GMQE	QMEAN	GMQE	QMEAN
O64411	0,98	0,19	0,54	-4,42	0,57	-3,69
A0A0D3H627	0,7	-0,43	0,51	-5,67	0,53	-5,02
A0A0D3HCW1	0,73	-0,56	0,55	-4,72	0,57	-4,39
A0A0D9XIZ7	0,78	-0,26	0,55	-5,84	0,56	-4,52
A0A0D9XIZ8	0,76	-1,91	0,54	-4,66	0,56	-3,31
A0A0D9XJ20	0,66	-0,89	0,46	-5,12	0,48	-3,93
A0A0E0M0H0	0,86	-0,45	0,54	-6,31	0,57	-5,31
A0A0E0M0H1	0,8	-1,53	0,54	-5,2	0,55	-5,73
A0A0E0M0H2	0,82	-1,52	0,54	-5,21	0,56	-5,47
A0A0E0MLW4	0,76	-0,53	0,52	-6,96	0,54	-4,88
B8BGH2	0,73	-0,72	0,54	-4,48	0,56	-4,32
D7TDE4	0,73	-0,47	0,55	-4,78	0,56	-4,71
D7TDE5	0,71	0,06	0,54	-4,32	0,55	-5,09
D7TDE8	0,78	-0,44	0,55	-6,09	0,57	-4,46
F2DTW7	0,81	-0,54	0,55	-5,08	0,56	-5,2
I1I2U3	0,72	-0,22	0,53	-5,5	0,56	-3,67
I1I3T4	0,73	-0,69	0,55	-5,54	0,57	-5,1
J3LKX2	0,87	-0,17	0,54	-5,2	0,57	-4,71
J3LKX3	0,73	-0,23	0,54	-5,5	0,56	-4,76
K4A8M0	0,7	-1,47	0,52	-6,1	0,54	-5,57
K4A8M6	0,71	-0,26	0,52	-6,26	0,22	-2,65
K4A8T5	0,72	-0,26	0,54	-5,29	0,56	-4,74
K4A8X1	0,72	-0,71	0,54	-5,27	0,56	-4,76
K4A901	0,83	0	0,55	-4,94	0,57	-4,42
K7TH00	0,98	0,2	0,54	-4,64	0,57	-3,81
M0VYV1	0,78	-0,5	0,54	-6,41	0,6	-4,44
M0XC59	0,83	-0,3	0,56	-4,8	0,57	-4,95
M7YHU7	0,78	-1,9	0,53	-6,77	0,55	-5,24
M7ZHC5	0,83	-0,32	0,57	-3,59	0,59	-2,73
Q0J291	0,68	-0,58	0,49	-5,33	0,51	-3,9
Q6H5M8	0,76	-0,51	0,55	-5	0,57	-5,1
Q93WC0	0,78	-0,41	0,54	-5,68	0,59	-4,39
Q93WM8	0,77	-0,16	0,54	-5,29	0,57	-5,17
T1MB50	0,79	-0,72	0,54	-5,19	0,56	-6,84
W5E475	0,82	-1,47	0,53	-5,88	0,56	-4,87
W5HZB1	0,81	-0,64	0,54	-5,3	0,56	-5,42
W5I2Y4	0,78	-0,66	0,54	-5,88	0,57	-4,48

Clade 3	ZmPAO1		FMS1		MmAPAO	
	GMQE	QMEAN	GMQE	QMEAN	GMQE	QMEAN
Q9SU79	0.53	-7.52	0.52	-6.77	0,61	-2,66
A0A022QPJ5	0.55	-5.81	0.53	-6.52	0,64	-3,43
A0A059CNL9	0.53	-6.31	0.50	-6.90	0,59	-4,41
A0A061GKP9	0.53	-6.53	0.49	-6.18	0,58	-3,72
A0A067F3K9	0.53	-6.55	0.49	-6.87	0,58	-4,42
A0A067KFR9	0.51	-7.49	0.49	-6.33	0,59	-3,55
A0A078EXI3	0.53	-6.29	0.51	-5.42	0,61	-2,83
A0A078GXG7	0.56	-6.29	0.55	-5.87	0,64	-3,69
A0A078I0C3	0.55	-6.58	0.53	-6.70	0,63	-3,35
A0A078IW94	0.55	-5.98	0.55	-5.62	0,63	-3,39
A0A087GI24	0.53	-6.73	0.51	-6.43	0,6	-4,15
A0A0A0LFR4	0.55	-5.69	0.53	-6.00	0,63	-3,72
A0A0B0N8V3	0.53	-6.91	0.50	-5.89	0,59	-3,5
A0A0D2PT72	0.55	-6.97	0.53	-5.94	0,63	-3,64
A0A0D2RT44	0.53	-6.61	0.50	-5.80	0,58	-4,08
A2Q567	0.54	-5.79	0.52	-6.02	0,62	-3,13
B9RK67	0.51	-6.27	0.48	-7.68	0,57	-3,54
F6HGW1	0.55	-7.42	0.54	-5.30	0,62	-3,66
I1L1H1	0.48	-6.09	0.46	-6.81	0,56	-4,38
I1MSH2	0.54	-5.94	0.52	-6.07	0,63	-2,9
K4BZE2	0.55	-5.47	0.54	-5.91	0,64	-3,26
K4CDQ7	0.55	-6.62	0.55	-5.99	0,64	-3,6
K7MBX1	0.50	-6.70	0.49	-7.53	0,58	-4,45
M1BIG2	0.54	-7.04	0.53	-6.44	0,63	-3,63
M1BXE0	0.55	-6.10	0.54	-5.76	0,63	-4,18
M1DUC0	0.53	-6.48	0.53	-6.74	0,6	-3,43
M5VPD8	0.51	-7.32	0.51	-6.65	0,58	-4,29
Q84XG2	0.53	-6.66	0.52	-6.24	0,61	-3,79
R0F4R6	0.53	-6.35	0.52	-6.25	0,6	-3,75
V4MCU1	0.50	-6.66	0.51	-6.47	0,6	-3,8
V4UKF2	0.52	-5.98	0.48	-6.89	0,58	-4,39
V7B1I2	0.51	-6.10	0.50	-7.35	0,59	-4,77
V7CBE3	0.53	-6.25	0.52	-6.16	0,61	-3,85
W9QJP6	0.51	-6.34	0.49	-6.06	0,58	-3,63

Clade 4	ZmPAO1		FMS1		MmAPAO	
	GMQE	QMEAN	GMQE	QMEAN	GMQE	QMEAN
A0A022R2Z4	0,57	-4,6	0,56	-3,62	0,58	-2,99
A0A022RSV7	0,57	-3,44	0,57	-5,35	0,57	-3,22
A0A059ADP6	0,58	-3,98	0,56	-4,51	0,57	-2,94
A0A059AEF1	0,57	-3,41	0,56	-3,82	0,57	-3,12
A0A059DGU2	0,57	-3,07	0,56	-4,35	0,57	-2,89
A0A061DK84	0,58	-2,95	0,56	-4,5	0,58	-2,84
A0A061DLV8	0,58	-3,22	0,56	-4,69	0,57	-3,67
A0A061DSU8	0,54	-4,65	0,52	-5,99	0,53	-4,48
A0A067EQD2	0,57	-3,56	0,56	-3,86	0,57	-3,46
A0A067KL71	0,58	-3,53	0,56	-3,83	0,58	-1,74
A0A067KNR0	0,58	-3,44	0,57	-4,16	0,57	-3,32
A0A067KRY6	0,58	-3,41	0,57	-4,62	0,58	-3,06
A0A072UW35	0,57	-3,25	0,56	-4,77	0,58	-3,47
A0A078BXS8	0,58	-3,86	0,56	-4,65	0,57	-3,32
A0A078D8A2	0,58	-4,04	0,6	-4,43	0,6	-2,92
A0A078EAX4	0,51	-3,98	0,5	-4,5	0,5	-3,28
A0A078FPS0	0,56	-3,3	0,56	-3,66	0,57	-3,2
A0A078FTZ1	0,56	-4,57	0,55	-4,82	0,56	-3,6
A0A078GXZ4	0,56	-3,64	0,56	-4,5	0,56	-3,45
A0A078I134	0,59	-4,94	0,58	-5,78	0,61	-2,64
A0A078IRH4	0,59	-5,23	0,56	-4,43	0,57	-3,47
A0A078ITG3	0,57	-5,29	0,55	-4,99	0,55	-4,99
A0A078IYL5	0,59	-5,02	0,57	-3,68	0,57	-3,28
A0A087GZ44	0,57	-4,67	0,56	-5,42	0,57	-2,93
A0A087H4U1	0,58	-3,39	0,56	-4,26	0,58	-2,98
A0A087HFW9	0,59	-5,13	0,57	-4,35	0,58	-3,02
A0A0A0KYS9	0,57	-2,91	0,56	-4,38	0,58	-2,58
A0A0A0KZ60	0,57	-4,4	0,57	-3,86	0,58	-3,21
A0A0D2RCX0	0,58	-3,26	0,57	-4,3	0,58	-3,16
A0A0D2RTI4	0,53	-8,99	0,5	-8,5	0,5	-7,24
A0A0D2T3J4	0,58	-3,33	0,57	-4,17	0,58	-2,7
A0A0D3G0K0	0,54	-5,79	0,54	-4,79	0,56	-3,92
A0A0D3G1I1	0,56	-3,1	0,56	-4,15	0,56	-3,39
A0A0D3G1I2	0,58	-2,85	0,56	-4,98	0,57	-3,24

Table S2: Continued.

Clade 4	ZmPAO1		FMS1		MmAPAO	
	GMQE	QMEAN	GMQE	QMEAN	GMQE	QMEAN
A0A0D9W9Q3	0,58	-3,91	0,57	-3,53	0,59	-2,7
A0A0D9WAZ4	0,58	-2,96	0,57	-3,88	0,58	-2,58
A0A0D9WAZ5	0,57	-3,39	0,56	-5,39	0,57	-3,83
A0A0D9ZQW3	0,55	-4,83	0,55	-4,14	0,56	-3,66
A0A0D9ZS90	0,57	-3,02	0,57	-2,95	0,57	-3,21
A0A0D9ZS91	0,57	-3,07	0,57	-4,2	0,57	-3,26
A0A0E0KVF6	0,58	-4,01	0,56	-4,82	0,59	-2,84
A0A0E0KWL7	0,54	-4,47	0,52	-6,02	0,54	-4,2
A0A0E0KWL8	0,57	-4,13	0,56	-4,81	0,55	-4,68
A0A0E0PE98	0,54	-5,79	0,54	-4,79	0,56	-3,92
A0A0E0PFI2	0,57	-3,15	0,56	-3,51	0,57	-3,03
A0A0E0PFI3	0,57	-3,07	0,57	-4,2	0,57	-3,26
A0A1D6DYV6	0,58	-4,01	0,57	-3,91	0,59	-2,88
A2XYT9	0,57	-3,02	0,57	-2,95	0,57	-3,21
B4F9F6	0,57	-3,45	0,57	-3,32	0,58	-2,3
B6SV76	0,56	-3,31	0,56	-4,11	0,56	-3,09
B6SYR8	0,58	-4,01	0,57	-4,25	0,59	-2,89
B8ARE0	0,57	-3,05	0,56	-5,2	0,57	-3,71
B8AUI2	0,58	-4,13	0,57	-4,06	0,59	-2,77
B9GSQ8	0,54	-5,12	0,54	-5,02	0,56	-3,95
B9H3J5	0,59	-5,27	0,56	-5,96	0,59	-2,94
B9H864	0,57	-2,54	0,56	-5,08	0,58	-2,72
B9S6G9	0,57	-3,24	0,55	-4,95	0,58	-2,67
B9SJL5	0,58	-5,79	0,55	-6,2	0,58	-2,95
C5YA47	0,57	-3,49	0,57	-3,17	0,57	-2,17
C5YA49	0,57	-3,17	0,58	-3,27	0,57	-3,08
C5YG61	0,58	-4,44	0,56	-4,34	0,58	-2,73
D7TDQ5	0,57	-4,43	0,57	-3,72	0,57	-3,39
E0CTZ8	0,58	-2,78	0,57	-4,07	0,58	-2,7
F2DFX4	0,58	-3,63	0,57	-4,03	0,58	-2,73
G7J7X8	0,57	-4,27	0,57	-3,73	0,58	-2,61
G7KD02	0,57	-3,59	0,58	-3,03	0,57	-3,8
I1J1Z5	0,58	-3,95	0,56	-4,67	0,59	-3,04
I1J380	0,57	-2,82	0,57	-2,98	0,57	-3,22
I1J381	0,57	-3,13	0,56	-4,72	0,57	-3,77
I1JJ00	0,57	-3,82	0,56	-5,1	0,58	-2,94
I1KXW2	0,57	-3,83	0,56	-4,36	0,58	-3
I1LM64	0,57	-4,18	0,58	-5,31	0,59	-2,71
I1M719	0,57	-3,94	0,56	-3,91	0,58	-2,77
I1MBQ3	0,57	-3,19	0,57	-4,37	0,57	-3,46
I1MZJ1	0,58	-4,78	0,56	-5,14	0,59	-2,93
I1N129	0,57	-3,51	0,56	-3,73	0,57	-3,08
I1PQ13	0,58	-4,21	0,57	-3,53	0,58	-2,68
I1PQS6	0,56	-3,04	0,56	-4,15	0,56	-3,39
I1PQS7	0,57	-2,84	0,56	-4,1	0,57	-3,38
J3M1J7	0,58	-4,11	0,56	-4,22	0,59	-2,87
J3M2I5	0,58	-3,35	0,57	-4,43	0,58	-2,95
J3M2I6	0,57	-3,29	0,57	-3,65	0,57	-3,94
K3Y6Y2	0,57	-4,24	0,56	-4,42	0,58	-2,83
K3Z5Q7	0,57	-3,49	0,56	-4,99	0,58	-3,31
K3Z5R8	0,58	-3,28	0,58	-3,02	0,58	-2,17
K4BA09	0,57	-3,29	0,57	-4,38	0,57	-2,83
K4BFF9	0,59	-3,98	0,59	-3,99	0,6	-2,25
K4CEL6	0,57	-3,31	0,57	-4,02	0,59	-2,25
K4DBH4	0,57	-3,06	0,58	-4,03	0,58	-3,06
K7KAF6	0,58	-2,93	0,58	-3,54	0,57	-3,07
K7TNR6	0,58	-4,16	0,57	-4,96	0,6	-2,57
M0RTD5	0,54	-3,66	0,53	-3,85	0,53	-3,42
M0SUJ5	0,56	-4,46	0,57	-3,8	0,57	-3,05
M0U284	0,58	-3,14	0,57	-3,7	0,58	-3,31
M0U7N9	0,57	-2,88	0,56	-4,09	0,57	-2,86
M0VUD5	0,57	-3,12	0,57	-4,21	0,58	-2,46
M1BAZ8	0,57	-3,57	0,56	-3,89	0,58	-2,47
M1BQU1	0,57	-3,81	0,57	-4,29	0,57	-2,91
M1BQU3	0,57	-3,83	0,54	-4,49	0,57	-2,93
M1C867	0,59	-3,92	0,59	-3,77	0,6	-2,88
M1CT69	0,59	-3,43	0,6	-4,03	0,59	-4,73
M1CT70	0,58	-3,81	0,58	-4,22	0,59	-3,96
M5WYW2	0,57	-4,59	0,57	-5,65	0,58	-3,95
M7Z7G8	0,58	-3,91	0,56	-4,85	0,58	-2,68

Clade 4	ZmPAO1		FMS1		MmAPAO	
	GMQE	QMEAN	GMQE	QMEAN	GMQE	QMEAN
M7ZYQ4	0,54	-4,23	0,54	-5,35	0,54	-5,29
Q0J954	0,57	-3,05	0,56	-5,2	0,57	-3,71
Q7X809	0,58	-4,21	0,57	-3,53	0,58	-2,68
Q7XR46	0,57	-3,02	0,57	-2,95	0,57	-3,21
Q8H191	0,58	-4,21	0,56	-5,47	0,57	-3,24
Q9LYT1	0,58	-3,83	0,58	-4,53	0,58	-3,31
Q9SKX5	0,57	-3,77	0,56	-4,32	0,57	-3,2
R0FMX3	0,58	-3,44	0,57	-4,42	0,58	-3,33
R0FWK2	0,57	-4,15	0,56	-4,55	0,56	-3,78
R0HP27	0,57	-4,13	0,56	-4,56	0,57	-3,94
R0HYV7	0,58	-5,44	0,56	-4,38	0,57	-2,68
S8E4F7	0,6	-3,1	0,59	-3,78	0,6	-2,87
U5FLG7	0,56	-4,49	0,55	-3,94	0,55	-3,97
V4KBX8	0,58	-5,33	0,56	-4,2	0,58	-2,56
V4LNX9	0,62	-4,99	0,6	-4,8	0,61	-3,24
V4LR45	0,59	-3,67	0,59	-3,99	0,6	-3,61
V4M9N7	0,57	-3,68	0,56	-4,25	0,57	-3,14
V4MK60	0,57	-3,66	0,57	-4,24	0,58	-2,98
V4SS93	0,57	-3,85	0,56	-4	0,57	-3,14
V4TFE5	0,54	-3,83	0,53	-5,24	0,55	-3,2
V4VD50	0,57	-2,75	0,57	-3,42	0,56	-2,8
V7B6B3	0,57	-3,91	0,56	-4,75	0,58	-3,58
V7B817	0,57	-3,75	0,57	-3,44	0,57	-2,63
V7BLV1	0,57	-3,47	0,57	-2,97	0,58	-3,14
V7BNY1	0,57	-3,6	0,57	-3,77	0,58	-2,92
W5AQH0	0,57	-3,13	0,57	-3,66	0,58	-2,2
W5ASS6	0,57	-3,32	0,56	-4,21	0,57	-3,66
W5BA09	0,57	-2,57	0,57	-3,59	0,57	-2,2
W5BWG6	0,57	-3,38	0,56	-3,77	0,57	-3,44
W9RTP2	0,6	-4,09	0,57	-4,12	0,58	-2,77

Clade 5	ZmPAO1		FMS1		MmAPAO	
	GMQE	QMEAN	GMQE	QMEAN	GMQE	QMEAN
A0A0D9V4T9	0.58	-6.00	0.58	-5.70	0,65	-3,03
A0A0D9YDX7	0.57	-6.37	0.57	-5.49	0,63	-2,74
A0A0E0JN44	0.58	-5.89	0.56	-5.91	0,63	-2,7
A2WUB8	0.57	-6.37	0.57	-5.49	0,63	-2,74
C0PE40	0.57	-4.90	0.55	-6.05	0,64	-3
C5XI79	0.56	-6.61	0.56	-6.24	0,64	-2,96
Q5NAI7	0.57	-6.36	0.56	-5.39	0,63	-3,12

Clade 6	ZmPAO1		FMS1		MmAPAO	
	GMQE	QMEAN	GMQE	QMEAN	GMQE	QMEAN
P55826	0,44	-7,75	0,44	-9,16	0,45	-7,2
Q9AR38	0,44	-6,87	0,04	0,56	0,44	-6,66
A0A022S294	0,44	-6,56	0,45	-6,64	0,45	-5,42
A0A058ZY55	0,44	-6,47	0,44	-8,48	0,45	-6,75
A0A061DRW0	0,44	-6,8	0,44	-8,73	0,44	-6,71
A0A067K7V8	0,43	-7,2	0,43	-8,94	0,44	-6,03
A0A072VNG3	0,42	-7,96	0,43	-8,28	0,43	-7,33
A0A078DL58	0,44	-6,48	0,44	-8,31	0,45	-6,92
A0A078FSF5	0,44	-6,99	0,44	-8,34	0,45	-6,67
A0A087GLJ5	0,44	-6,49	0,44	-8,13	0,45	-6,02
A0A0A0KMZ2	0,43	-7,05	0,43	-8	0,43	-6,85
A0A0D2SID4	0,44	-6,04	0,45	-8,24	0,45	-6,12
A0A0D9UZU6	0,42	-8	0,43	-9,06	0,43	-9,12
A0A0D9UZU7	0,45	-6,75	0,45	-8,98	0,46	-6,18
A0A0D9UZU8	0,42	-7,31	0,28	-6,43	0,43	-8,08
A0A0E0JH98	0,41	-7,89	0,43	-8,84	0,43	-6,81
A0A0E0MUV5	0,44	-7,15	0,45	-7,43	0,44	-7,41
A0A0E0MUV6	0,42	-7,76	0,43	-8,72	0,43	-6,94
A0A0R4J461	0,44	-6,65	0,43	-8,73	0,44	-6,15
B8ACN2	0,44	-7,55	0,44	-8,47	0,44	-6,54
B9GTA7	0,43	-7,55	0,44	-8,26	0,44	-6,64
B9I9A1	0,41	-7,32	0,41	-7,91	0,42	-6,3
B9RN34	0,44	-7,3	0,44	-8,87	0,45	-6,35
C0P5U0	0,43	-6,83	0,44	-7,43	0,44	-6,66
C5XHZ0	0,45	-5,89	0,45	-7,28	0,45	-6,63
D7U2T4	0,43	-6,07	0,44	-7,72	0,43	-5,79
I1HEP2	0,45	-7,59	0,46	-6,8	0,45	-6,74
I1NMB9	0,44	-7,23	0,44	-8,17	0,44	-6,43
K3XGF3	0,44	-6,89	0,45	-7,73	0,45	-6,78

Table S2: Continued.

Clade 6	ZmPAO1		FMS1		MmAPAO	
	GMQE	QMEAN	GMQE	QMEAN	GMQE	QMEAN
K4AXF5	0,44	-6,99	0,43	-9,07	0,42	-6
M0RSM0	0,44	-7,03	0,44	-8,21	0,44	-6,32
M0ZKS8	0,42	-7,52	0,43	-6	0,42	-7,17
M5X9A2	0,43	-6,87	0,43	-9,22	0,44	-6,07
R0GTZ0	0,44	-6,81	0,44	-7,81	0,45	-6,68
R0H9S5	0,44	-6,76	0,44	-8,09	0,45	-6,65
S8DH59	0,44	-7,78	0,44	-9,03	0,45	-5,53
V4U1E8	0,43	-7,15	0,44	-8,32	0,44	-6,68
V4VWZ0	0,44	-7,14	0,43	-8,94	0,43	-7
W5FMA5	0,45	-6,79	0,46	-7,93	0,46	-5,5
W9QNJ2	0,43	-6,65	0,43	-6	0,45	-5,64

Clade 7	ZmPAO1		FMS1		MmAPAO	
	GMQE	QMEAN	GMQE	QMEAN	GMQE	QMEAN
Q8S9J1	0,46	-6,76	0,48	-6,14	0,51	-6,25
A0A022RWF9	0,44	-7,8	0,45	-6,87	0,47	-7,45
A0A022RXS4	0,45	-5,39	0,43	-6,36	0,47	-7,21
A0A022RYU5	0,44	-7,31	0,45	-7,14	0,48	-6,44
A0A059AH03	0,43	-7,45	0,43	-8,21	0,45	-7,77
A0A061ECM9	0,49	-8,02	0,49	-5,44	0,52	-6,98
A0A067LD55	0,42	-6,42	0,47	-6,81	0,5	-6,68
A0A078BZ14	0,47	-6,69	0,47	-7,75	0,51	-6,68
A0A078J522	0,47	-7,2	0,47	-7,18	0,5	-6,75
A0A0A0K7K1	0,5	-6,64	0,53	-6,21	0,53	-6
A0A0D2S037	0,49	-7,42	0,48	-6,89	0,51	-7,02
A0A0D2S042	0,46	-7,34	0,45	-7,56	0,48	-6,64
A0A0D2VX85	0,45	-7,14	0,45	-7,54	0,48	-6,48
A0A0D2VX88	0,49	-7,14	0,48	-6,65	0,52	-6,8
A0A0E0PAV8	0,44	-5,36	0,35	-6,3	0,45	-5,02
A0A0K9PTR4	0,48	-6,96	0,48	-6,74	0,49	-6,16
B4F9N9	0,44	-5,91	0,44	-6,49	0,45	-7,2
B8AVU8	0,46	-6,05	0,44	-8,56	0,47	-6,4
B9RBL4	0,46	-7,34	0,47	-7,31	0,5	-7,42
C5YBC2	0,44	-6,61	0,44	-6,54	0,45	-7,5
D7UBE4	0,47	-7,88	0,47	-8,02	0,51	-7,06
G7L3C0	0,48	-6,68	0,49	-5,8	0,52	-6,96
I1IZ42	0,47	-7,08	0,47	-6,91	0,47	-7,84
I1PMI3	0,47	-5,6	0,47	-6,63	0,49	-6,4
J3LZ44	0,44	-5,45	0,44	-6	0,44	-7,42
K3Y6C6	0,45	-5,81	0,45	-6,19	0,46	-7,6
K4BDT2	0,48	-6,47	0,47	-8,03	0,51	-7,27
M0RIS9	0,42	-6	0,42	-7,03	0,44	-7,98
M1B1J6	0,49	-7,32	0,48	-7,82	0,52	-6,51
M5X092	0,5	-6,95	0,48	-8,4	0,54	-6,11
Q7X7T4	0,47	-6,32	0,47	-7,53	0,49	-6,31
Q9SLW5	0,47	-7,85	0,48	-6,65	0,51	-7,73
R0H9J9	0,41	-8,5	0,42	-8,02	0,44	-6
V4U383	0,47	-6	0,46	-7,94	0,49	-7,48
V4UDK6	0,47	-6,46	0,47	-6,74	0,5	-6,69
W5AQ00	0,44	-7,42	0,44	-6	0,45	-7,29
W9SIW4	0,49	-6,75	0,5	-5,17	0,51	-6

Clade 8	ZmPAO1		FMS1		MmAPAO	
	GMQE	QMEAN	GMQE	QMEAN	GMQE	QMEAN
Q8S4R4	0,4	-5,98	0,38	-7,66	0,4	-6,62
Q9M9Y8	0,41	-6,98	0,38	-8,14	0,41	-5,71
A0A022RFY7	0,39	-7,89	0,38	-9,67	0,39	-6,13
A0A067G549	0,36	-7,64	0,38	-5,62	0,37	-5,91
A0A067G5N7	0,37	-6,04	0,35	-7,18	0,35	-7,04
A0A078F054	0,4	-8,24	0,39	-8,29	0,41	-6,35
A0A078FAA4	0,41	-7,5	0,48	-10,43	0,41	-5,98
A0A087HLJ4	0,41	-6,82	0,39	-7,44	0,41	-5,73
A0A0D9XTE9	0,4	-6,58	0,39	-8,5	0,4	-6,73
A0A0E0MGH9	0,44	-5,2	0,41	-7,64	0,43	-6,37
A0A0K9Q298	0,39	-8,36	0,39	-7,47	0,39	-7
B8BL57	0,38	-7,78	0,18	-5,84	0,38	-7,48
B9IIH0	0,4	-7,18	0,38	-8,12	0,39	-5,3
C1JFF5	0,41	-7,33	0,39	-6,93	0,4	-6,5
C5Y4W1	0,41	-7,69	0,4	-6,43	0,41	-5,65
I1H789	0,41	-6,91	0,39	-6,38	0,41	-5,44
I1IKZ2	0,41	-8,35	0,4	-6,92	0,41	-5,67
I1R124	0,41	-7,28	0,39	-8,89	0,41	-5,36

Clade 8	ZmPAO1		FMS1		MmAPAO	
	GMQE	QMEAN	GMQE	QMEAN	GMQE	QMEAN
J3N949	0,45	-6,75	0,44	-7,47	0,45	-6,97
K3ZHU6	0,41	-6,79	0,4	-7,35	0,4	-6,44
M0XMD8	0,41	-7,33	0,39	-6,68	0,41	-5,81
M1CQH9	0,4	-5,78	0,38	-6	0,39	-5,52
Q0IS25	0,4	-5,98	0,38	-6,18	0,39	-5,13
R0IBW2	0,41	-6,24	0,39	-7,7	0,4	-5,98
V4KEQ2	0,4	-6,19	0,39	-7,3	0,41	-5,45
V4SRC3	0,36	-6,46	0,35	-7,95	0,36	-6,1
V7BII3	0,38	-5,34	0,36	-7,72	0,37	-5,3
W5A9A2	0,41	-6	0,39	-7,52	0,4	-6,33
W9STZ4	0,39	-7,19	0,38	-7,47	0,39	-5,51

Clade 9	ZmPAO1		FMS1		MmAPAO	
	GMQE	QMEAN	GMQE	QMEAN	GMQE	QMEAN
A0A022QAA2	0,46	-6,45	0,44	-8,45	0,46	-6,33
A0A022QFY7	0,42	-6,67	0,4	-8,14	0,41	-6,83
A0A059B8W2	0,42	-5,87	0,39	-7,87	0,4	-6,76
A0A059B9U2	0,42	-6,15	0,4	-7,72	0,41	-7,08
A0A061E424	0,39	-7,29	0,38	-7,85	0,38	-6,48
A0A067L5E5	0,42	-6,42	0,4	-8,02	0,41	-6,5
A0A072UUM0	0,41	-7,4	0,4	-7,92	0,41	-6,65
A0A078E1M5	0,42	-7,05	0,41	-8,37	0,42	-5,03
A0A078HN33	0,41	-7,33	0,4	-8,26	0,4	-6,51
A0A0A0L3B1	0,41	-7,27	0,4	-8,51	0,41	-6,04
A0A0B0P8R7	0,38	-6,88	0,35	-8,51	0,37	-7,43
A0A0D2NR92	0,42	-6,58	0,4	-8,66	0,41	-6,48
A0A0D2QA89	0,42	-7,05	0,4	-6	0,41	-6,35
A0A0D9W181	0,4	-6,86	0,39	-7,15	0,39	-6,22
A0A0D9ZFL9	0,4	-7,36	0,39	-6,41	0,4	-6,28
A0A0E0KLE5	0,4	-6,7	0,39	-7,61	0,4	-5,13
A0A0E0P3Q7	0,4	-7,08	0,39	-6,91	0,4	-6,58
A0A0K9PWG9	0,42	-6,54	0,39	-9,07	0,41	-6,44
A2XNW6	0,41	-7,09	0,39	-7,31	0,4	-6,91
B9SKB8	0,42	-6,98	0,4	-7,96	0,41	-7,12
C5WT16	0,41	-6,92	0,38	-7,92	0,4	-6,91
I1GL11	0,4	-7,56	0,38	-7,77	0,39	-6,53
I1KNT0	0,42	-7,52	0,41	-9,05	0,42	-8,07
I1PH84	0,43	-7,15	0,41	-7,6	0,42	-6,9
J3LUN4	0,43	-8,17	0,42	-6	0,43	-6,23
K4A7D7	0,41	-6,84	0,38	-8,44	0,4	-6,49
K4BXJ1	0,42	-7,03	0,41	-7,82	0,42	-5,83
M0TNP6	0,46	-6,98	0,43	-8,69	0,45	-6,73
M1CCL6	0,43	-7,16	0,41	-6	0,42	-6,55
M5XHL3	0,42	-7,64	0,4	-8,65	0,41	-6,38
Q10AT7	0,41	-7,09	0,39	-7,63	0,4	-7,19
Q9FVR9	0,42	-7,31	0,4	-8,59	0,41	-8,02
R0IE47	0,43	-6,94	0,41	-8,25	0,42	-6,28
U7DZJ4	0,43	-6,82	0,41	-8,4	0,42	-5,94
V4KIE4	0,42	-6,87	0,4	-9,66	0,42	-6,21
V4KQP4	0,42	-7,21	0,41	-7,82	0,41	-6,75
V4T5K4	0,42	-7,95	0,4	-7,61	0,41	-6,72
V7C6V4	0,43	-8,08	0,41	-9,2	0,43	-6,51
W9R9Z5	0,41	-7,37	0,38	-8,95	0,4	-6,61

Clade 10	ZmPAO1		FMS1		MmAPAO	
	GMQE	QMEAN	GMQE	QMEAN	GMQE	QMEAN
A0A061DXU5	0,43	-6,09	0,2	-6	0,42	-6,47
B9H1K7	0,42	-6,23	0,48	-8,35	0,41	-6,49
B9S5J6	0,41	-6,97	0,21	-5,39	0,42	-6,86
F6I0Y5	0,44	-7,06	0,41	-8,52	0,41	-7,45
M5VLW0	0,43	-7,49	0,21	-4,34	0,42	-6,56
W9QIW2	0,42	-6,55	0,22	-5,61	0,41	-6,83

Clade 11	ZmPAO1		FMS1		MmAPAO	
	GMQE	QMEAN	GMQE	QMEAN	GMQE	QMEAN
A0A022PQC8	0,51	-4,8	0,48	-6,09	0,47	-5,36
A0A059AT55	0,49	-4,59	0,46	-7,14	0,47	-5,12
A0A061E2J9	0,47	-5,89	0,46	-7,66	0,47	-5,41
A0A067DBI6	0,46	-5,41	0,32	-5,53	0,44	-5,66
A0A072UNG5	0,47	-5,73	0,47	-7,89	0,47	-5,16
A0A078HSS6	0,47	-6,72	0,47	-6,57	0,46	-5,96
A0A078I4A4	0,47	-6,04	0,46	-6,32	0,47	-5,5
A0A087H899	0,47	-5,76	0,48	-5,53	0,47	-5,2

Table S2: Continued.

	ZmPAO1		FMS1		MmAPAO	
Clade 11	GMQE	QMEAN	GMQE	QMEAN	GMQE	QMEAN
A0A0A0LCJ0	0,48	-5,87	0,46	-6,11	0,47	-5,35
A0A0D2TPG3	0,47	-5,6	0,47	-7,07	0,47	-4,97
A0A0D9X1R2	0,47	-6,9	0,48	-6,09	0,48	-5,1
A0A0E0AMZ3	0,47	-6,79	0,47	-6	0,47	-5,8
A0A0E0LNL2	0,45	-6	0,44	-6,94	0,45	-5,36
A0A0E0QBJ3	0,47	-6,98	0,47	-7,22	0,47	-5,82
B8B4V7	0,47	-6,83	0,48	-6,61	0,47	-5,66
B9RNM3	0,47	-6	0,45	-7,55	0,46	-6,25
C5X2L2	0,47	-7,2	0,48	-6,39	0,48	-5,26
D7ST93	0,48	-6,43	0,47	-6	0,48	-5,37
I1GS51	0,47	-6,43	0,47	-6,42	0,47	-5,63
I1QCE6	0,47	-5,93	0,47	-6,57	0,48	-5,36
K3ZZJ7	0,48	-5,85	0,47	-6,7	0,47	-4,91
M0SGK6	0,48	-6,81	0,47	-6,18	0,47	-4,93
M0ZW80	0,47	-5,82	0,46	-5,41	0,46	-6,05
Q8LI32	0,47	-6,98	0,47	-7,22	0,47	-5,82
Q9SF45	0,47	-5,64	0,47	-6,27	0,46	-5,82
R0I6N3	0,47	-5,93	0,47	-6,7	0,46	-6,2
V4LBV6	0,48	-5,13	0,47	-6,3	0,47	-4,9
V4V1Q1	0,52	-5,16	0,49	-6,75	0,49	-6,06
V7CQ56	0,47	-5,79	0,45	-7,98	0,46	-5,39
W5BME1	0,47	-7,35	0,46	-7,03	0,47	-5,78
W9SB51	0,47	-5,29	0,45	-6,55	0,46	-6,07

	ZmPAO1		FMS1		MmAPAO	
Clade 12	GMQE	QMEAN	GMQE	QMEAN	GMQE	QMEAN
Q38893	0,41	-8,45	0,05	-3,11	0,4	-8,25
Q9SE20	0,4	-7,61	0,05	-2,19	0,4	-7,38
Q9ZTP4	0,4	-7,33	0,4	-8,92	0,4	-7,33
A0A022Q1Z0	0,42	-8,06	0,06	-1,82	0,41	-8,35
A0A022Q702	0,41	-7,41	0,4	-8,23	0,41	-7,22
A0A058ZTR3	0,4	-7,9	0,05	-1,65	0,39	-7,5
A0A059B6A2	0,4	-7,77	0,05	-1,66	0,39	-8,03
A0A061F1B6	0,41	-6,69	0,05	-1,75	0,39	-7,95
A0A067JUH5	0,4	-7,57	0,38	-8,59	0,38	-8,84
A0A072VMI4	0,39	-8,09	0,06	-2,43	0,39	-7,26
A0A078HTK0	0,41	-7,34	0,05	-1,67	0,4	-8,22
A0A0A0M100	0,4	-7,17	0,05	-1,8	0,4	-7,7
A0A0B0N2Q2	0,41	-7,15	0,05	-1,68	0,4	-8,07
A0A0B0NU44	0,41	-7,81	0,06	-1,13	0,4	-8,1
A0A0D2TN81	0,41	-8,47	0,05	-1,2	0,4	-8,69
A0A0D2VHZ9	0,4	-7,72	0,05	-1,29	0,4	-8,5
A0A0D3GNH4	0,44	-8,11	0,06	-2,91	0,44	-6,74
A0A0E0LIB4	0,4	-8,04	0,39	-9,13	0,4	-7,46
A0A0E0Q5E4	0,4	-8,41	0,39	-8,71	0,4	-7,04
A0A0K9PSZ6	0,43	-7,48	0,06	-2,29	0,42	-8,34
B8B894	0,41	-8,29	0,04	-2,16	0,4	-7,07
B9I8C7	0,4	-7,63	0,05	-3,22	0,39	-6
D7TUM8	0,42	-7,71	0,06	-1,18	0,41	-8,87
G3DR91	0,41	-7,57	0,06	-3,28	0,41	-7,71
G7I6X1	0,4	-8,23	0,39	-8,28	0,4	-7
I1H2R4	0,41	-8,81	0,4	-9,63	0,4	-7,6
I1LBJ5	0,41	-7,8	0,05	-1,6	0,4	-7,26
I1NIT4	0,4	-8,37	0,06	-0,9	0,4	-7,55
I1Q8Y4	0,4	-7,79	0,39	-8,7	0,4	-6,96
J3MJC5	0,44	-7,61	0,06	-2,92	0,43	-8,1
K3ZRV6	0,4	-8,28	0,05	-0,88	0,4	-7,64
M0SPM6	0,41	-7,95	0,05	-3,25	0,4	-7,63
M1C1T5	0,4	-8,14	0,05	-2,17	0,4	-7,27
M5WAB3	0,06	-2,25	0,06	-2,25	0,4	-7,76
M8B1I5	0,44	-8,19	0,42	-9,73	0,43	-7,14
Q0D7W4	0,4	-8,41	0,39	-8,71	0,4	-7,04
Q202I0	0,4	-7,81	0,05	-2,2	0,4	-7,14
Q7XB60	0,41	-8,29	0,4	-8,54	0,4	-7,07
R0G4E0	0,41	-8,18	0,06	-1,55	0,42	-7,66
S8DET4	0,46	-7,36	0,07	-1,78	0,45	-8,3
U5GE99	0,4	-7,42	0,38	-8,3	0,39	-7,33
V4M3X9	0,4	-7,83	0,06	-1,88	0,4	-8,86
V7BJQ1	0,4	-8,07	0,05	-1,96	0,39	-7,41
W9QKC9	0,4	-7,5	0,05	-1,54	0,38	-8,76

	ZmPAO1		FMS1		MmAPAO	
Clade 13	GMQE	QMEAN	GMQE	QMEAN	GMQE	QMEAN
A2XDA1	0,43	-5,29	0,41	-7,9	0,42	-6
P28553	0,43	-5,48	0,4	-8,35	0,41	-6,36
P28554	0,41	-5,57	0,4	-7,69	0,41	-6,5
P49086	0,43	-5,64	0,41	-7,46	0,42	-7,1
Q07356	0,44	-6,28	0,42	-7,62	0,42	-7,47
Q0DUI8	0,43	-5,29	0,41	-7,9	0,42	-6
A0A022Q293	0,42	-6,01	0,41	-6,83	0,42	-6,68
A0A059B1A4	0,42	-5,84	0,41	-6,98	0,41	-6,94
A0A061ERY4	0,44	-5,68	0,43	-7,5	0,43	-6,76
A0A067K8K2	0,41	-6,26	0,41	-7,44	0,41	-6,58
A0A078FIC9	0,44	-5,55	0,42	-7,09	0,43	-6,96
A0A078JBN5	0,43	-5,53	0,42	-7,49	0,42	-6,45
A0A0A0KVZ4	0,43	-5,79	0,41	-8,41	0,43	-5,63
A0A0B0NZ61	0,41	-7,13	0,04	-3,35	0,4	-7,82
A0A0D2VMJ7	0,44	-6,33	0,42	-8,04	0,43	-6
A0A0D3FEP6	0,42	-5,81	0,2	-7,31	0,41	-7,33
A0A0D9VQE0	0,42	-5,77	0,41	-7,95	0,41	-6,93
A0A0D9Z321	0,41	-5,88	0,2	-6,68	0,4	-6,98
A0A0E0K9R7	0,41	-5,89	0,21	-5,55	0,41	-6,51
A0A0E0LS19	0,43	-4,82	0,41	-7,27	0,42	-5,31
A0A0E0NQN0	0,43	-5,29	0,41	-7,9	0,42	-6,53
A0A0E0NQN1	0,42	-5,72	0,41	-7,9	0,41	-6,4
A0A0K9PMA5	0,41	-5,73	0,4	-7,76	0,41	-6,7
B8APP7	0,44	-5,89	0,43	-7,33	0,43	-6,76
B9GND3	0,42	-5,93	0,41	-7,32	0,41	-7,27
B9IAB4	0,42	-6,14	0,41	-7,56	0,41	-7,08
B9RY09	0,4	-6,33	0,39	-7,7	0,4	-6,5
C4J008	0,43	-5,38	0,41	-8,16	0,42	-7,14
C4PW00	0,42	-5,46	0,41	-7,69	0,41	-6,52
D7TZI5	0,42	-5,67	0,41	-7,37	0,41	-7,53
F4JUN0	0,44	-6,28	0,42	-7,62	0,42	-7,47
I1H8Y3	0,43	-4,87	0,41	-7,63	0,41	-6,11
I1LN47	0,43	-5,46	0,41	-7,72	0,42	-6,55
I1MYC3	0,42	-5,77	0,41	-7,89	0,42	-6,26
I1MYC4	0,39	-4,25	0,3	-5,52	0,31	-4,52
J3LKP8	0,42	-5,29	0,41	-8,43	0,41	-7,05
K4A7Q8	0,43	-5,15	0,41	-7,84	0,42	-6,5
M0U8M7	0,42	-5,44	0,41	-7,12	0,42	-5,92
M0UGD2	0,41	-5,86	0,4	-8,02	0,41	-6,97
M1AIS6	0,43	-5,88	0,4	-8,06	0,41	-6,67
M5XQ45	0,43	-5,74	0,41	-7,61	0,47	-9,26
M7YC32	0,46	-5,99	0,44	-9,12	0,45	-6,2
R0F4I7	0,44	-5,78	0,42	-8,17	0,43	-7,01
S8CPB8	0,47	-5,67	0,45	-7,68	0,45	-7,07
V4LZV7	0,44	-6,01	0,43	-6,93	0,43	-6,82
V7D270	0,42	-6,46	0,4	-8,85	0,42	-5,94
W5DWR8	0,42	-5,11	0,4	-8,56	0,41	-6,08
W5EL23	0,42	-5,58	0,41	-8,27	0,41	-5,72

Table S2: Model quality parameters. GMQE and QMEAN of models obtained with three templates for each sequence within each clade. ZmPAO1 (pdb code 3KU9), FMS1 (pdb code 1XPQ) and MmAPAO (pdb code 5MBX).

Table S3

		SignalP 4.1	WoLF PSORT	LOCALIZER			slplocal2	DeepLoc1,0	PredSL	TargetP1.1	Pprowler
Name	clade	Signal Score	Loc Score	Chloroplast	Mitochondria	Nucleus	Loc SVM score	Loc Score	prediction	Loc Score	Loc Score
G7J0U8	I	N0.268	plas:7.5	-	-	-	nucleus_or_cytosol0.39478146	Cytoplasm0.8533	chloroplast	S4	OTHER0.77
A0A072UW15	I	N0.268	plas:7.5	-	-	-	nucleus_or_cytosol0.06622016	Cytoplasm0.7221	chloroplast	S4	OTHER0.63
K7LI87	I	N0.255	plas:7.5	-	-	-	secretory_pathway0.45107200	Cytoplasm0.6843	secreted	S3	OTHER0.74
V7BV40	I	N0.298	vacu:5	-	-	-	nucleus_or_cytosol0.05446361	Cytoplasm0.5787	secreted	S3	OTHER0.49
K7ME99	I	N0.257	plas:8	-	-	-	nucleus_or_cytosol0.21333403	Cytoplasm0.7346	secreted	S3	OTHER0.60
B9RVL4	I	N0.325	E.R.:5.5	-	-	-	secretory_pathway0.48809790	Cytoplasm0.8176	chloroplast	S3	OTHER0.72
L7VGD5	I	N0.280	extr:3	-	-	-	nucleus_or_cytosol0.00977270	Cytoplasm0.8368	secreted	S3	OTHER0.53
A0A0D2R6C7	I	N0.282	E.R.:4.5	-	-	-	nucleus_or_cytosol0.15928194	Cytoplasm0.8492	secreted	S3	SP0.41
A0A061DW64	I	N0.244	vacu:4	-	-	-	nucleus_or_cytosol0.26176697	Cytoplasm0.8521	chloroplast	S3	OTHER0.56
A0A061E2Z7	I	N0.244	vacu:4	-	-	-	nucleus_or_cytosol0.25927044	Cytoplasm0.8498	chloroplast	S3	OTHER0.52
A0A0D2QT21	I	N0.318	E.R.:4.5	-	-	-	secretory_pathway0.28884167	Cytoplasm0.8483	chloroplast	S2	SP0.42
A0A0D2RUK9	I	N0.318	E.R.:5.5	-	-	-	secretory_pathway0.28107034	Cytoplasm0.8457	chloroplast	S2	OTHER0.54
V4TFW4	I	N0.204	extr:4	-	-	-	nucleus_or_cytosol0.18095351	Cytoplasm0.8724	secreted	S4	OTHER0.86
A0A0A0K600	I	N0.275	vacu:4	-	-	-	nucleus_or_cytosol0.21281705	Cytoplasm0.871	chloroplast	S4	OTHER0.82
D7T359	I	N0.252	vacu:3	-	-	-	nucleus_or_cytosol0.36865403	Cytoplasm0.6138	secreted	S3	OTHER0.79
M1A995	I	N0.387	vacu:7	-	-	-	nucleus_or_cytosol-0.08592746	Cytoplasm0.4442	secreted	S4	OTHER0.81
K4AYE9	I	N0.364	vacu:8	-	-	-	nucleus_or_cytosol-0.00076017	Cytoplasm0.4998	secreted	S5	OTHER0.81
Q4H439	I	Y0.471	vacu:8	-	-	-	secretory_pathway-0.16795579	Peroxisome0.371	secreted	S3	OTHER0.58
A0A059BIZ8	I	N0.311	vacu:6	-	-	-	nucleus_or_cytosol0.52682817	Cytoplasm0.532	secreted	S3	OTHER0.78
A0A059BJ23	I	N0.356	vacu:6	-	-	-	secretory_pathway0.13542218	Cytoplasm0.4974	secreted	S3	OTHER0.68
M5X3Z6	I	N0.259	plas:9.5	-	-	-	secretory_pathway0.29404245	Cytoplasm0.8589	secreted	S3	OTHER0.74
Q0PCS5	I	N0.242	E.R.:4.5	-	-	-	nucleus_or_cytosol0.28799324	Cytoplasm0.8361	chloroplast	S3	OTHER0.73
S8EIZ2	I	N0.332	extr:5	-	-	-	secretory_pathway0.64348852	Cytoplasm0.7447	secreted	S3	SP0.63
A0A022RVP1	I	N0.422	extr:4	-	-	-	nucleus_or_cytosol0.62525321	Cytoplasm0.457	secreted	S3	OTHER0.74
I1L5L5	I	N0.312	plas:10	-	-	-	nucleus_or_cytosol0.35931991	Cytoplasm0.5456	secreted	S3	OTHER0.64
Q9FNA2	I	Y0.454	vacu:4	-	-	-	secretory_pathway0.85824741	Cytoplasm0.7984	secreted	S2	OTHER0.54
V4LC82	I	N0.337	chlo:5	-	-	-	secretory_pathway0.88823709	Cytoplasm0.8549	secreted	S3	OTHER0.73
R0FM29	I	N0.111	nucl:12.5	-	-	Y(RRERDSRERRDNRDLERRDRD RSPLPPPRRDYKRRPSLSPPPPY RDRRRGGNRGG)	chloroplast0.71288662	Nucleus0.4734	chloroplast	C3	OTHER0.78
Cs7g02060	I	N0.204	extr: 4	-	-	-	nucleus_or_cytosol0.18095351	Cytoplasm0.8724	secreted	S4	OTHER0.86
I1I2U3	II	Y0.711	chlo:10	Y (0.984 | 1-32)	-	-	secretory_pathway0.38676630	Extracellular0.4698	secreted	S1	SP0.99
W5I2Y4	II	Y0.742	chlo:11	-	Y (0.8 | 1-31)	-	secretory_pathway0.32157377	Extracellular0.4453	secreted	S2	SP0.99
Q93WC0	II	Y0.705	chlo:9	-	-	-	secretory_pathway0.44407093	Extracellular0.6179	secreted	S1	SP0.99
M0VYV1	II	Y0.705	chlo:9	-	-	-	secretory_pathway0.43790411	Extracellular0.0061	secreted	S1	SP0.99
K4A8M0	II	Y0.699	chlo:4	-	-	-	secretory_pathway0.88138298	Extracellular0.6613	secreted	S1	SP0.99
K4A8T5	II	Y0.704	vacu:4	-	-	-	secretory_pathway0.93224680	Extracellular0.6458	secreted	S1	SP0.99
K4A8M6	II	Y0.704	vacu:4	-	-	-	secretory_pathway0.94316072	Extracellular0.6386	secreted	S1	SP0.99
K4A8X1	II	Y0.704	vacu:4	-	-	-	secretory_pathway0.82230092	Extracellular0.6073	secreted	S1	SP0.99
I1I3T4	II	Y0.778	vacu:5	-	-	-	secretory_pathway111277230	Extracellular0.6295	secreted	S1	SP1.00
M7ZHC5	II	Y0.796	chlo:4	-	-	-	mitochondria0.63748781	Extracellular0.4464	secreted	S2	SP0.82
W5HZB1	II	Y0.710	chlo:3	-	-	-	mitochondria0.35215017	Extracellular0.3695	secreted	S1	SP0.83
F2DTW7	II	Y0.790	chlo:3	-	-	-	secretory_pathway0.92926265	Extracellular0.3789	secreted	S1	SP0.99
M0XC59	II	Y0.735	chlo:4	-	-	-	secretory_pathway0.81255165	Extracellular0.5341	secreted	S1	SP0.90
B8BGH2	II	Y0.742	chlo:4	-	-	-	secretory_pathway107035880	Extracellular0.473	secreted	S1	SP0.98
A0A0D3HCW1	II	Y0.741	chlo:4	-	-	-	secretory_pathway110317130	Extracellular0.4879	secreted	S1	SP1.00
A0A0D9XJ20	II	Y0.805	chlo:5	-	-	-	secretory_pathway153951440	Extracellular0.4806	secreted	S1	SP1.00
J3LKX2	II	Y0.719	chlo:5	-	-	-	secretory_pathway0.75790030	Extracellular0.4497	secreted	S1	SP1.00
J3LKX3	II	Y0.578	chlo:8	-	-	-	secretory_pathway0.53716449	Extracellular0.5188	secreted	S1	SP0.99
K4A901	II	Y0.770	vacu:6	-	-	-	secretory_pathway0.72813521	Endoplasmic reticulum0.462	secreted	S1	SP1.00

Table S3: Continued

Name	clade	SignalP 4.1 Signal Score	WoLF PSORT Loc Score	LOCALIZER Chloroplast	LOCALIZER Mitochondria	LOCALIZER Nucleus	slplocal2 Loc SVM score	DeepLoc1,0 Loc Score	PredSL prediction	TargetP1.1 Loc Score	Pprowler Loc Score
K7TH00	II	Y0.830	chlo:7	Y (0.818 | 1-51)	-	-	secretory_pathway0.87128539	Endoplasmic reticulum0.3392	secreted	S1	SP0.99
O64411	II	Y0.843	chlo:7	Y (0.852 | 1-51)	-	-	secretory_pathway0.94029320	reticulum0.3573	secreted	S1	SP0.99
T1MB50	II	Y0.779	chlo:7	-	-	-	secretory_pathway0.75214902	Extracellular0.4949	secreted	S1	SP0.99
Q93WM8	II	Y0.728	chlo:8	Y (0.876 | 1-46)	-	-	secretory_pathway0.26587727	Extracellular0.6609	secreted	S1	SP0.99
W5E475	II	Y0.729	chlo:8	Y (0.903 | 1-51)	-	-	secretory_pathway0.42400426	Extracellular0.457	secreted	S1	SP0.99
M7YHU7	II	Y0.701	chlo:12	Y (0.881 | 1-41)	-	-	mitochondria0.50075740	Extracellular0.4129	secreted	S2	SP0.98
Q0J291	II	N0.177	cyto:6	-	-	-	secretory_pathway0.11544524	Endoplasmic reticulum0.7068	other	S3	SP0.92
Q6H5M8	II	Y0.721	chlo:13	-	-	-	secretory_pathway148147640	Extracellular0.5105	secreted	S3	SP0.99
A0A0D3H627	II	Y0.806	chlo:4	-	-	-	secretory_pathway0.52549958	Endoplasmic reticulum0.4898	secreted	S2	SP0.99
A0A0E0M0H0	II	N0.192	chlo:7	-	Y (0.975 | 1-21)	-	mitochondria107892990	Mitochondrion0.8234	mitochondrion	M2	MTP0.96
A0A0E0M0H1	II	Y0.764	chlo:6	-	Y (0.766 | 1-32)	-	secretory_pathway112793940	Endoplasmic reticulum0.4153	secreted	S1	SP0.99
A0A0E0M0H2	II	Y0.764	chlo:6	-	Y (0.766 | 1-32)	-	secretory_pathway112149780	Endoplasmic reticulum0.4186	secreted	S1	SP0.99
A0A0D9XIZ7	II	N0.127	cyto:10	-	-	Y (PKPS)	nucleus_or_cytosol-0.09146501	Cytoplasm0.7078	secreted	_3	SP0.02
A0A0D9XIZ8	II	N0.127	cyto:10	-	-	Y (PKPS)	nucleus_or_cytosol-0.08823335	Cytoplasm0.774	secreted	_3	SP0.02
A0A0E0MLW4	II	N0.124	chlo:6	-	-	-	nucleus_or_cytosol0.61632239	Cytoplasm0.5573	other	_4	SP0.02
D7TDE4	II	Y0.610	extr:5	-	-	Y(KKQEDISVLTAQRLKNR)	secretory_pathway0.58376597	Lysosome/Vacuole0.5021	secreted	S1	SP0.99
D7TDE5	II	N0.297	E.R.:4.5	-	-	-	secretory_pathway-0.27345114	Lysosome/Vacuole0.6395	secreted	S5	SP0.79
D7TDE8	II	Y0.765	chlo:5	-	Y (0.999 | 1-21)	-	mitochondria0.58119845	Endoplasmic reticulum0.497	mitochondrion	M4	SP0.94
A0A0D2RT44	III	N0.361	cyto:8	-	-	Y (KKPR)	secretory_pathway0.15749769	Peroxisome0.4154	secreted	S5	OTHER0.69
A0A0D2PT72	III	N0.345	cyto:5	-	-	Y (KKPR)	secretory_pathway0.24319407	Mitochondrion0.2394	secreted	S3	OTHER0.70
F6HGW1	III	N0.329	cyto:6	-	-	Y (KKPR)	secretory_pathway-0.03054257	Peroxisome0.457	secreted	S5	OTHER0.68
A0A067KFR9	III	N0.335	cyto:6	-	-	-	secretory_pathway0.42443263	Peroxisome0.3731	secreted	S4	OTHER0.75
B9RK67	III	N0.330	cyto:6	-	-	Y (KKPR)	secretory_pathway-0.18307370	Mitochondrion0.4507	secreted	M5	OTHER0.62
V4UKF2	III	N0.369	chlo:5	-	-	Y (KKPR)	secretory_pathway0.19954417	Mitochondrion0.4075	secreted	_5	OTHER0.68
A0A067F3K9	III	N0.369	chlo:5	-	-	Y (KKPR)	secretory_pathway0.19002592	Mitochondrion0.4002	secreted	_5	OTHER0.68
A0A059CNL9	III	N0.436	chlo:5	-	-	Y (KKPR)	secretory_pathway-0.12990371	Mitochondrion0.5147	secreted	M4	MTP0.50
W9QJP6	III	N0.335	cyto:4	-	-	Y (KKPR,KVKK)	secretory_pathway0.22808918	Peroxisome0.3914	secreted	S5	OTHER0.62
A2Q567	III	N0.256	cyto:5	-	-	Y (KVKK)	secretory_pathway0.67057689	Cytoplasm0.3578	secreted	S4	OTHER0.77
M1BIG2	III	N0.301	cyto:7	-	-	Y (KRRK)	secretory_pathway0.36156879	Cytoplasm0.4901	secreted	S3	OTHER0.61
K4CDQ7	III	N0.372	nucl:4	-	-	Y (KRRK,RRKR)	secretory_pathway0.50371166	Cytoplasm0.4063	secreted	S2	SP0.40
M1BXE0	III	N0.308	cyto:6	-	-	-	secretory_pathway0.21297444	Peroxisome0.4286	secreted	S5	OTHER0.59
K4BZE2	III	N0.318	cyto:7	-	-	Y (RHRR)	secretory_pathway0.19584566	Peroxisome0.4348	secreted	S3	OTHER0.62
A0A0A0LFR4	III	N0.301	nucl:6	-	-	-	secretory_pathway0.94198706	Peroxisome0.4246	secreted	S2	SP0.55
A0A022QPJ5	III	N0.239	cyto:7	-	-	Y (KKPR)	secretory_pathway0.05465223	Peroxisome0.4359	secreted	S3	OTHER0.64
A0A022RYW5	III	N0.259	cyto:6	-	-	-	secretory_pathway0.30367457	Peroxisome0.4734	secreted	M4	OTHER0.51
Q9SU79	III	N0.418	mito:3	-	-	-	secretory_pathway-0.07911158	Peroxisome0.4245	secreted	S4	OTHER0.46
R0F4R6	III	N0.418	cyto:3.5	-	-	-	secretory_pathway-0.09608699	Peroxisome0.5097	secreted	S3	OTHER0.38
V4MCU1	III	N0.345	cyto:5.5	-	-	Y (KKPR)	secretory_pathway-0.52521680	Peroxisome0.4136	secreted	S4	OTHER0.46
I1L1H1	III	N0.276	cyto:7	-	-	Y(KKPR,KKVTRIEWQLDDEKRKKGG)	secretory_pathway0.87572701	Peroxisome0.2799	secreted	S4	SP0.43
A0A087GI24	III	N0.422	chlo:5	-	Y (0.942 | 1-33)	-	secretory_pathway0.03056824	Mitochondrion0.4058	secreted	S4	OTHER0.38
Q84XG2	III	N0.254	cyto:5.5	-	-	Y (KKPR,KVKK)	secretory_pathway-0.45248963	Peroxisome0.4317	chloroplast	S4	OTHER0.43
A0A078I0C3	III	N0.337	cyto:5.5	-	-	Y (KKPR)	secretory_pathway-0.43969506	Mitochondrion0.4612	secreted	S5	OTHER0.40
A0A078GXG7	III	N0.322	cyto:5.5	-	Y (0.818 | 1-28)	Y (KKPR)	secretory_pathway-0.06391588	Mitochondrion0.5902	secreted	M5	MTP0.40
A0A078IW94	III	N0.359	cyto:5.5	-	-	Y (KKPR,RKKK)	secretory_pathway-0.40404998	Mitochondrion0.4087	secreted	S5	OTHER0.63
M1DUC0	III	N0.243	nucl:9	-	-	-	secretory_pathway0.42188941	Mitochondrion0.514	secreted	S5	OTHER0.69
K7MBX1	III	N0.276	cyto:8	-	-	Y(KKPR,RKVTRIEWQLDDEKRKGA)	secretory_pathway0.70852438	Peroxisome0.3051	secreted	S4	OTHER0.64
V7B1I2	III	N0.270	cyto:7	-	-	Y (KKPR)	secretory_pathway0.67097674	Mitochondrion0.3603	secreted	S4	OTHER0.62
I1MSH2	III	N0.412	chlo:8	-	-	-	secretory_pathway0.71969891	Mitochondrion0.2388	secreted	S2	OTHER0.48
V7CBE3	III	N0.403	chlo:4	-	-	-	secretory_pathway0.29520466	Mitochondrion0.4116	secreted	S4	OTHER0.39
M5VPD8	III	N0.313	cyto:5	-	-	-	secretory_pathway0.39856272	Peroxisome0.4083	secreted	S3	SP0.43
A0A061GKP9	III	N0.362	cyto:5	-	-	Y(KKPR,PTGKRKA)	secretory_pathway-0.08036100	Peroxisome0.4381	secreted	S5	SP0.37
A0A0B0N8V3	III	N0.361	chlo:5	-	-	Y (KKPR)	secretory_pathway0.22257799	Peroxisome0.4057	secreted	S5	OTHER0.55
Cs4g14150	III	N0.369	chlo:5	-	-	Y (KKPR)	secretory_pathway0.19954417	Mitochondrion0.4075	secreted	_5	OTHER0.58

Table S3: Continued

		SignalP 4.1	WoLF PSORT	LOCALIZER			slplocal2	DeepLoc1,0	PredSL	TargetP1.1	Pprowler
Name	clade	Signal Score	Loc Score	Chloroplast	Mitochondria	Nucleus	Loc SVM score	Loc Score	prediction	Loc Score	Loc Score
G7J7X8	IV	N0.147	pero:5	-	-	-	nucleus_or_cytosol0.00113676	Cytoplasm0.4836	secreted	_2	OTHER0.86
I1LM64	IV	N0.159	pero:7	-	-	-	nucleus_or_cytosol0.25695541	Cytoplasm0.5423	secreted	_2	OTHER0.87
I1MZJ1	IV	N0.139	pero:6	-	-	-	nucleus_or_cytosol0.16778546	Peroxisome0.443	secreted	_3	OTHER0.83
V7BLV1	IV	N0.156	pero:6	-	-	-	nucleus_or_cytosol-0.21995317	Peroxisome0.3854	secreted	_5	OTHER0.69
B9H3J5	IV	N0.170	pero:5	-	-	-	nucleus_or_cytosol0.73543096	Peroxisome0.4673	secreted	_3	OTHER0.88
A0A067KL71	IV	N0.151	pero:12	-	-	-	nucleus_or_cytosol0.45151478	Cytoplasm0.5383	chloroplast	_3	OTHER0.75
B9SJL5	IV	N0.118	plas:5	-	-	-	nucleus_or_cytosol0.35868751	Peroxisome0.4954	secreted	_2	OTHER0.89
V4SS93	IV	N0.119	pero:7	-	-	-	nucleus_or_cytosol0.50577658	Peroxisome0.5744	secreted	_2	OTHER0.85
A0A067EQD2	IV	N0.119	pero:7	-	-	-	nucleus_or_cytosol0.50125717	Peroxisome0.5837	secreted	_2	OTHER0.85
I1MBQ3	IV	N0.150	E.R.:7	-	-	-	nucleus_or_cytosol0.27997800	Cytoplasm0.4536	secreted	_2	OTHER0.90
K7KAF6	IV	N0.154	pero:6	-	-	-	nucleus_or_cytosol0.38148231	Peroxisome0.4291	other	_2	OTHER0.90
V7B817	IV	N0.152	E.R.:6	-	-	-	nucleus_or_cytosol0.53482518	Peroxisome0.6103	secreted	_2	OTHER0.92
M1BQU1	IV	N0.128	pero:5	-	-	-	nucleus_or_cytosol116525570	Peroxisome0.5343	other	_2	OTHER0.90
M1BQU3	IV	N0.122	pero:7	-	-	-	nucleus_or_cytosol0.95792098	Peroxisome0.5343	other	_2	OTHER0.90
K4BA09	IV	N0.125	pero:7	-	-	-	nucleus_or_cytosol105646820	Peroxisome0.5518	other	_2	OTHER0.91
M1C867	IV	N0.175	pero:6	-	-	-	nucleus_or_cytosol0.27742807	Peroxisome0.4589	secreted	M5	OTHER0.83
K4BFF9	IV	N0.160	pero:6	-	-	-	nucleus_or_cytosol0.35696768	Mitochondrion0.354	secreted	M5	OTHER0.88
D7TDQ5	IV	N0.137	pero:5	Y (0.996 | 1-28)	-	Y(RRRR)	mitochondria0.92162873	Peroxisome0.4695	chloroplast	M5	MTP0.60
A0A022R2Z4	IV	N0.107	pero:6	-	-	-	nucleus_or_cytosol144591160	Cytoplasm0.5504	other	_2	OTHER0.93
M5WYW2	IV	Y0.679	pero:5	-	-	-	secretory_pathway0.79109258	Extracellular0.5717	secreted	S2	SP0.99
S8D8B3	IV	N0.129	pero:12	-	-	-	nucleus_or_cytosol105931150	Cytoplasm0.6698	secreted	_2	OTHER0.90
A0A059ADP6	IV	N0.135	pero:12	-	-	-	nucleus_or_cytosol0.11133186	Cytoplasm0.5203	secreted	_2	OTHER0.90
A0A059AEF1	IV	N0.135	pero:12	-	-	-	nucleus_or_cytosol0.12084919	Cytoplasm0.5414	secreted	_2	OTHER0.90
Q8H191	IV	N0.142	pero:6	-	-	-	nucleus_or_cytosol147352490	Peroxisome0.5031	secreted	_3	OTHER0.93
R0HYV7	IV	N0.147	pero:6	-	-	-	nucleus_or_cytosol0.77963473	Peroxisome0.5332	secreted	_2	OTHER0.93
V4KBX8	IV	N0.142	pero:6	-	-	-	nucleus_or_cytosol0.70059143	Peroxisome0.5554	secreted	_3	OTHER0.92
A0A087HFW9	IV	N0.167	pero:5	-	-	-	nucleus_or_cytosol0.04228207	Peroxisome0.5072	secreted	_4	OTHER0.80
A0A078FTZ1	IV	N0.153	pero:6	-	-	-	nucleus_or_cytosol0.60919150	Peroxisome0.4946	secreted	_2	OTHER0.91
A0A078ITG3	IV	N0.152	pero:7	-	-	-	nucleus_or_cytosol0.64868920	Cytoplasm0.4911	secreted	_2	OTHER0.92
I1J380	IV	N0.154	E.R.:6	-	-	Y (AAAED)	nucleus_or_cytosol0.13779734	Cytoplasm0.5739	secreted	_5	OTHER0.76
W5AQH0	IV	N0.143	chlo:7	-	-	-	nucleus_or_cytosol0.15125620	Cytoplasm0.6663	secreted	_5	OTHER0.76
W5BA09	IV	N0.143	chlo:7	-	-	-	nucleus_or_cytosol0.15170482	Cytoplasm0.6676	secreted	_5	OTHER0.76
M0VUD5	IV	N0.144	chlo:7	Y (0.896 | 1-26)	-	-	nucleus_or_cytosol-0.11620091	Cytoplasm0.6238	secreted	_5	OTHER0.65
I1PQS6	IV	N0.148	chlo:11	-	-	-	nucleus_or_cytosol0.10352621	Cytoplasm0.6683	secreted	_4	OTHER0.79
A0A0D3G1I1	IV	N0.148	chlo:11	-	-	-	nucleus_or_cytosol0.10352621	Cytoplasm0.6683	secreted	_4	OTHER0.79
A2XYT9	IV	N0.148	chlo:11	-	-	-	nucleus_or_cytosol0.10489332	Cytoplasm0.675	secreted	_4	OTHER0.79
Q7XR46	IV	N0.148	chlo:11	-	-	-	nucleus_or_cytosol0.10489332	Cytoplasm0.675	secreted	_4	SP0.51
A0A0D9ZS90	IV	N0.148	chlo:11	-	-	-	nucleus_or_cytosol0.10489332	Cytoplasm0.675	secreted	_4	OTHER0.51
A0A0E0PFI2	IV	N0.148	chlo:11	-	-	-	nucleus_or_cytosol0.11106280	Cytoplasm0.6744	secreted	_4	SP/OTHER0.35
A0A0E0KWL7	IV	N0.147	chlo:8	-	-	-	nucleus_or_cytosol0.19517261	Cytoplasm0.5864	secreted	_4	SP0.40
J3M2I5	IV	N0.147	chlo:11	-	-	-	nucleus_or_cytosol0.05737362	Cytoplasm0.4999	secreted	_4	OTHER0.46
A0A0D9WAZ4	IV	N0.142	chlo:11	-	-	-	nucleus_or_cytosol0.16975125	Cytoplasm0.6306	secreted	_4	OTHER0.68
K3Z5R8	IV	N0.148	chlo:8	-	-	Y (AAAED)	nucleus_or_cytosol0.14483126	Cytoplasm0.5622	secreted	_4	OTHER0.84
B4F9F6	IV	N0.144	chlo:9	-	-	-	nucleus_or_cytosol0.12693013	Cytoplasm0.4834	secreted	_4	OTHER0.77
C5YA47	IV	N0.145	chlo:11	-	-	-	nucleus_or_cytosol0.11911062	Cytoplasm0.5204	secreted	_5	OTHER0.77
I1J381	IV	N0.132	pero:11	-	-	-	nucleus_or_cytosol0.57045856	Cytoplasm0.5781	other	_3	OTHER0.89
M7ZYQ4	IV	N0.132	pero:11	-	-	-	nucleus_or_cytosol0.64578639	Cytoplasm0.6053	other	_3	OTHER0.89
W5ASS6	IV	N0.129	pero:10	-	-	-	nucleus_or_cytosol0.59355406	Cytoplasm0.5485	other	_3	OTHER0.89
W5BWG6	IV	N0.124	pero:10	-	-	-	nucleus_or_cytosol0.59684863	Cytoplasm0.5286	other	_3	OTHER0.89

Table S3: Continued

		SignalP 4.1	WoLF PSORT	LOCALIZER			slplocal2	DeepLoc1,0	PredSL	TargetP1.1	Pprowler
Name	clade	Signal Score	Loc Score	Chloroplast	Mitochondria	Nucleus	Loc SVM score	Loc Score	prediction	Loc Score	Loc Score
I1PQS7	IV	N0.141	pero:11	-	-	-	nucleus_or_cytosol0.23158240	Cytoplasm0.5552	other	_3	OTHER0.83
A0A0D3G1I2	IV	N0.141	pero:10	-	-	-	nucleus_or_cytosol0.22987184	Cytoplasm0.543	other	_3	OTHER0.83
B8ARE0	IV	N0.141	pero:11	-	-	-	nucleus_or_cytosol0.38477158	Cytoplasm0.5803	other	_3	OTHER0.84
Q0J954	IV	N0.141	pero:11	-	-	-	nucleus_or_cytosol0.38477158	Cytoplasm0.5803	other	_3	OTHER0.84
A0A0D9ZS91	IV	N0.141	pero:10	-	-	-	nucleus_or_cytosol0.38708325	Cytoplasm0.566	other	_3	OTHER0.83
A0A0E0PFI3	IV	N0.141	pero:10	-	-	-	nucleus_or_cytosol0.38708325	Cytoplasm0.566	other	_3	OTHER0.83
J3M2I6	IV	N0.143	pero:10	-	-	-	nucleus_or_cytosol0.49119039	Cytoplasm0.5204	other	_3	OTHER0.86
A0A0E0KWL8	IV	N0.141	pero:9	-	-	-	nucleus_or_cytosol0.38280486	Cytoplasm0.57	other	_3	OTHER0.83
A0A0D9WAZ5	IV	N0.134	pero:11	-	-	-	nucleus_or_cytosol0.53458647	Cytoplasm0.5709	other	_3	OTHER0.87
K3Z5Q7	IV	N0.123	pero:6	-	-	Y (AAAED)	nucleus_or_cytosol0.94153228	Cytoplasm0.5059	other	_3	OTHER0.90
C5YA49	IV	N0.129	pero:5	-	-	Y (AAAED)	nucleus_or_cytosol0.78542931	Cytoplasm0.4527	secreted	_3	OTHER0.90
B6SV76	IV	N0.130	pero:6	-	-	Y (AAAED)	nucleus_or_cytosol0.76177094	Peroxisome0.486	secreted	_3	OTHER0.90
M0U7N9	IV	N0.126	pero:5	-	Y (0.868 | 1-22)	-	mitochondria0.06101865	Mitochondrion0.4552	secreted	_5	MTP0.79
M0U284	IV	N0.120	pero:8	-	-	-	mitochondria-0.05997662	Peroxisome0.3347	other	M5	MTP0.72
A0A0D2RTI4	IV	N0.202	E.R.:5	-	-	-	nucleus_or_cytosol0.14093689	Cytoplasm0.6504	other	_3	OTHER0.89
G7KD02	IV	N0.124	pero:4	-	-	-	mitochondria0.23991670	Peroxisome0.5348	secreted	_4	OTHER0.78
I1JJ00	IV	N0.119	pero:5	-	-	-	mitochondria0.48976097	Peroxisome0.5711	secreted	_5	OTHER0.49
I1M719	IV	N0.122	pero:4	-	-	-	mitochondria0.74414814	Peroxisome0.3612	mitochondrion	M4	MTP0.65
V7B6B3	IV	N0.130	pero:10	-	-	-	mitochondria0.50057783	Peroxisome0.6122	secreted	_4	SP0.45
I1KXW2	IV	N0.140	pero:8	-	-	-	mitochondria0.28510739	Peroxisome0.648	secreted	M5	MTP0.57
V7BNY1	IV	N0.124	pero:9	-	-	-	mitochondria100590310	Peroxisome0.6418	mitochondrion	M3	MTP0.90
A0A072UW35	IV	N0.129	pero:4	-	-	-	mitochondria0.04296839	Peroxisome0.7601	secreted	_4	MTP0.50
B9GSQ8	IV	N0.136	cyto:3	-	-	-	mitochondria0.84825823	Peroxisome0.5839	secreted	M5	MTP0.53
B9H864	IV	N0.137	pero:4	-	-	-	mitochondria0.69734886	Peroxisome0.5972	mitochondrion	M3	MTP0.78
A0A067KNR0	IV	N0.120	pero:9	-	-	-	mitochondria0.92096230	Peroxisome0.142	secreted	M5	MTP0.58
B9S6G9	IV	N0.122	pero:9	-	Y (0.821 | 1-22)	-	mitochondria105620700	Peroxisome0.5265	other	M3	MTP0.96
A0A067KRY6	IV	N0.150	chlo:6	-	-	-	mitochondria132364490	Peroxisome0.4109	secreted	M3	MTP0.57
V4TFE5	IV	N0.156	E.R.:6	-	Y (0.964 | 1-21)	-	mitochondria110786220	Mitochondrion0.3921	mitochondrion	M2	MTP0.82
V4VD50	IV	N0.156	E.R.:6	-	Y (0.964 | 1-21)	-	mitochondria106873170	Peroxisome0.3923	mitochondrion	M2	MTP0.97
A0A061DK84	IV	N0.156	plas:6	-	-	Y(RRGLCYSNGARRQAR)	mitochondria134907920	Peroxisome0.5424	other	M4	MTP0.89
A0A061DLV8	IV	N0.156	E.R.:6	-	-	Y(RRGLCYSNGARRQAR)	mitochondria132195580	Peroxisome0.5379	other	M4	MTP0.77
A0A061DSU8	IV	N0.156	plas:7	-	-	Y(RRGLCYSNGARRQAR)	mitochondria132600400	Peroxisome0.5494	other	M4	MTP0.78
A0A0D2RCX0	IV	N0.137	plas:7.5	-	-	-	nucleus_or_cytosol0.41098370	Peroxisome0.3881	other	_3	OTHER0.77
A0A0D2T3J4	IV	N0.137	plas:8.5	-	-	-	nucleus_or_cytosol0.40729720	Peroxisome0.3957	other	_3	OTHER0.76
E0CTZ8	IV	N0.136	pero:7	-	-	-	mitochondria124160610	Peroxisome0.652	secreted	M4	MTP0.77
A0A059DGU2	IV	N0.173	pero:4	-	-	-	mitochondria0.03428240	Peroxisome0.4375	chloroplast	M4	MTP0.77
M1BAZ8	IV	N0.139	pero:5	-	-	-	mitochondria0.42183070	Peroxisome0.5861	chloroplast	M4	MTP0.83
K4CEL6	IV	N0.139	pero:5	-	-	-	mitochondria0.44067745	Peroxisome0.5834	chloroplast	M4	MTP0.64
M1CT69	IV	N0.216	chlo:3	-	-	-	nucleus_or_cytosol-0.37210443	Mitochondrion0.4491	secreted	M2	SP0.43
M1CT70	IV	N0.138	pero:4	-	-	-	mitochondria0.05543279	Peroxisome0.4043	chloroplast	M5	SP0.56
K4DBH4	IV	N0.136	pero:5	-	-	-	mitochondria0.08030951	Peroxisome0.3875	chloroplast	C5	MTP0.61
S8E4F7	IV	N0.166	chlo:3	-	-	-	nucleus_or_cytosol-0.10064861	Peroxisome0.4415	secreted	M4	SP0.54
A0A022RSV7	IV	N0.125	pero:7	-	-	-	nucleus_or_cytosol-0.21455901	Peroxisome0.5555	chloroplast	_3	OTHER0.39
W9RTP2	IV	N0.120	pero:8	-	-	-	mitochondria0.64018900	Peroxisome0.6094	chloroplast	C5	MTP0.50
Q9LYT1	IV	N0.127	pero:10	-	-	Y(RKAICVSTDEKMKKKRSP)	nucleus_or_cytosol0.40920761	Peroxisome0.4914	other	_3	OTHER0.69
R0FMX3	IV	N0.122	pero:10	-	-	Y(RKAICVSTEEKMKKS)	mitochondria0.14320960	Peroxisome0.5115	other	_3	OTHER0.55
V4LNX9	IV	N0.334	pero:7	-	-	-	nucleus_or_cytosol-0.50725874	Peroxisome0.3629	secreted	S4	MTP0.49
A0A087GZ44	IV	N0.133	pero:12	-	-	-	nucleus_or_cytosol0.70190878	Peroxisome0.4839	secreted	_2	OTHER0.91
Q9SKX5	IV	N0.130	pero:13	-	-	Y(RRANCFSAGERMKTR)	mitochondria0.97644114	Peroxisome0.625	secreted	_5	MTP0.56

Table S3: Continued

		SignalP 4.1	WoLF PSORT		LOCALIZER		slplocal2	DeepLoc1,0	PredSL	TargetP1.1	Pprowler
Name	clade	Signal Score	Loc Score	Chloroplast	Mitochondria	Nucleus	Loc SVM score	Loc Score	prediction	Loc Score	Loc Score
R0FWK2	IV	N0.130	pero:13	-	-	Y(RRANCFSAGERMKTR)	mitochondria101646060	Peroxisome0.5973	secreted	_5	MTP0.57
R0HP27	IV	N0.143	pero:12	-	Y (0.701 | 1-52)	Y(RKNSDRQMRRAGERMKTR)	mitochondria143014700	Peroxisome0.5681	other	_5	OTHER0.66
V4LR45	IV	N0.215	pero:4	-	-	-	nucleus_or_cytosol-0.25424336	Peroxisome0.479	other	S5	OTHER0.59
V4M9N7	IV	N0.124	pero:7	-	-	Y(RRANCFSAGERMKTR)	mitochondria0.91708696	Peroxisome0.591	secreted	M5	MTP0.57
V4MK60	IV	N0.136	pero:7	-	Y (0.723 | 1-42)	Y(RKNSDRQMRRAGERMKTR)	mitochondria133242840	Peroxisome0.556	other	_5	OTHER0.66
A0A078FPS0	IV	N0.135	pero:13	-	Y (0.865 | 1-47)	Y(RRANCFSAGGRMKTR)	mitochondria0.96391100	Peroxisome0.6008	other	M5	MTP0.66
A0A078GXZ4	IV	N0.135	pero:13	-	Y (0.865 | 1-47)	Y(RRANCFSAGGRMKTR)	mitochondria100963090	Peroxisome0.5923	other	M5	MTP0.66
A0A078IRH4	IV	N0.138	pero:13	-	Y (0.741 | 1-42)	Y(RRANCFSAGGRMKTR)	mitochondria125159810	Peroxisome0.5329	other	M5	MTP0.77
A0A078IYL5	IV	N0.141	pero:13	-	Y (0.773 | 1-42)	Y(RRANCFSAGGRMKTR)	mitochondria120729690	Peroxisome0.5364	other	M4	MTP0.78
A0A087H4U1	IV	N0.121	pero:12	-	-	Y(RRANCFSASERMKER)	mitochondria0.82885375	Peroxisome0.6069	secreted	_5	MTP0.48
A0A078I134	IV	N0.146	pero:11	-	-	-	mitochondria0.88341359	Peroxisome0.513	secreted	_5	OTHER0.59
A0A0A0KZ60	IV	N0.139	E.R.:7	-	-	-	mitochondria0.35589672	Peroxisome0.5873	other	_5	MTP0.51
A0A0A0KYS9	IV	N0.128	pero:13	-	-	-	nucleus_or_cytosol0.48048142	Peroxisome0.6315	other	_3	OTHER0.79
I1J1Z5	IV	N0.147	pero:4	-	-	-	nucleus_or_cytosol0.43613063	Peroxisome0.4873	secreted	C5	OTHER0.63
M7Z7G8	IV	N0.135	pero:9	-	-	-	nucleus_or_cytosol0.65575247	Peroxisome0.5081	secreted	C5	OTHER0.61
F2DFX4	IV	N0.135	pero:9	-	-	-	nucleus_or_cytosol0.64979648	Peroxisome0.5015	secreted	C5	OTHER0.61
I1PQ13	IV	N0.147	cyto:3	-	-	-	nucleus_or_cytosol0.36951682	Peroxisome0.5928	secreted	M4	OTHER0.48
Q7X809	IV	N0.147	cyto:3	-	-	-	nucleus_or_cytosol0.36951682	Peroxisome0.5928	secreted	M4	OTHER0.48
B8AUI2	IV	N0.147	cyto:3	-	-	-	nucleus_or_cytosol0.37344479	Peroxisome0.6136	secreted	M4	OTHER0.48
A0A0E0KVF6	IV	N0.119	cyto:3	-	-	-	nucleus_or_cytosol0.53722336	Peroxisome0.5951	secreted	M4	OTHER0.74
J3M1J7	IV	N0.147	cyto:3	-	-	-	nucleus_or_cytosol0.36706839	Peroxisome0.5903	secreted	M4	OTHER0.48
A0A0D9W9Q3	IV	N0.144	pero:5	-	-	-	nucleus_or_cytosol0.55815667	Peroxisome0.5961	secreted	M5	OTHER0.74
A0A0D3G0K0	IV	N0.147	cyto:3	-	-	-	nucleus_or_cytosol0.16850484	Peroxisome0.5592	secreted	M4	OTHER0.49
A0A0E0PE98	IV	N0.147	cyto:3	-	-	-	nucleus_or_cytosol0.16850484	Peroxisome0.5592	secreted	M4	OTHER0.49
A0A0D9ZQW3	IV	N0.120	pero:4	-	-	-	nucleus_or_cytosol0.23317338	Peroxisome0.5723	secreted	M5	OTHER0.65
K3Y6Y2	IV	N0.161	pero:4	-	-	-	nucleus_or_cytosol0.40851823	Peroxisome0.5461	secreted	M5	OTHER0.70
B6SYR8	IV	N0.159	pero:9	-	-	-	nucleus_or_cytosol0.25195057	Peroxisome0.5105	secreted	M4	OTHER0.74
A0A1D6DYV6	IV	N0.147	pero:9	-	-	-	nucleus_or_cytosol0.45219347	Peroxisome0.5389	secreted	M5	OTHER0.71
C5YG61	IV	N0.150	pero:5	-	-	-	nucleus_or_cytosol0.39175282	Peroxisome0.5612	secreted	M5	OTHER0.73
K7TNR6	IV	N0.188	pero:5	-	-	-	nucleus_or_cytosol0.05284737	Peroxisome0.5178	secreted	_5	OTHER0.61
M0RTD5	IV	N0.147	cyto:3	-	-	-	mitochondria0.54431166	Mitochondrion0.3849	mitochondrion	M4	MTP0.76
M0SUJ5	IV	N0.109	pero:13	-	-	-	nucleus_or_cytosol0.76211000	Peroxisome0.4629	secreted	_3	OTHER0.84
Cs6g15870	IV	N0.156	E.R.:6	-	Y (0.964 | 1-21)	-	mitochondria110786220	Peroxisome0.5685	mitochondrion	M2	MTP0.98
Cs7g18840	IV	N0.119	pero:7	-	-	-	nucleus_or_cytosol0.56336096	Mitochondrion0.3921	secreted	_2	OTHER0.85
I1NR63	V	N0.335	cyto:9	-	-	Y (KKPR)	nucleus_or_cytosol0.10407777	Peroxisome0.6499	secreted	S5	OTHER0.56
A2WUB8	V	N0.335	cyto:8	-	-	Y (KKPR)	nucleus_or_cytosol-0.28047196	Peroxisome0.6832	secreted	S5	OTHER0.56
A0A0D9YDX7	V	N0.335	cyto:8	-	-	Y (KKPR)	nucleus_or_cytosol-0.28047196	Peroxisome0.6832	secreted	S5	OTHER0.56
Q5NAI7	V	N0.335	cyto:9	-	-	Y (KKPR)	nucleus_or_cytosol0.05755408	Peroxisome0.6408	secreted	S5	OTHER0.56
A0A0E0JN44	V	N0.355	chlo:7	-	-	Y (KRPR)	mitochondria-0.39851706	Peroxisome0.5904	secreted	M4	MTP0.71
A0A0D9V4T9	V	N0.320	chlo:7	-	-	Y (KRVK)	nucleus_or_cytosol-0.13108212	Peroxisome0.6938	secreted	S5	MTP0.47
C5XI79	V	N0.329	cyto:8	-	-	Y (KKPR)	nucleus_or_cytosol-0.24018258	Peroxisome0.5554	secreted	M5	OTHER0.54
C0PE40	V	N0.283	chlo:8	-	-	-	nucleus_or_cytosol-0.33675529	Peroxisome0.5223	secreted	M4	MTP0.65

Table S4: Prediction of subcellular location. Sequences were analyzed with SignalP 4.1 (cbs.dtu.dk/services/SignalP/), WoLF PSORT (wolfpsort.hgc.jp), LOCALIZER (localizer.csiro.au/), slplocal2 (sunflower.kuicr.kyoto-u.ac.jp/~smatsuda/slplocal.html), DeepLoc1,0 (cbs.dtu.dk/services/DeepLoc/), PredSL (aias.biol.uoa.gr/PredSL/), TargetP1.1 (cbs.dtu.dk/services/TargetP/) and Pprowler (bioinf.scmb.uq.edu.au:8080/pprowler_webapp_1-2/).

Table S4

Subfamily 1

ZmPAO1	MmAPAO	A0A022RVP1	A0A059BIZ8	A0A059BJ23	A0A061DW64	A0A061E2Z7	A0A072UW15	A0A078C0U9	A0A078CR66	A0A0A0K600	A0A0D2QT21	A0A0D2R6C7	A0A0D2RUK9	B9GFC0	B9RVL4	D7T359	G7J0U8
Tyr 437	Thr 469	Y	Y	Y	Y	Y	Y	Y	Y	Y	-	Y	Y	Y	Y	Y	Y
Phe 401	-	Y	Y	Y	Y	Y	Y	Y	Y	Y	Y	Y	Y	Y	Y	Y	Y
-	Cys 181	-	-	-	-	-	-	-	-	-	-	-	-	-	-	-	-
-	Asn 308	-	-	-	-	-	-	-	-	-	-	-	-	-	-	-	-
Glu 168	Val 182	E	E	E	E	E	E	E	E	E	E	E	E	E	E	E	E
Tyr 296	Tyr 425	Y	Y	Y	Y	Y	Y	Y	Y	Y	Y	Y	Y	Y	Y	Y	Y
Glu 60	His 59	A	V	M	A	A	A	A	A	V	A	A	A	A	A	A	A
Tyr 167	Tyr 199	F	F	I	F	F	F	F	F	F	F	F	F	F	F	F	F
Tyr 163	Ser 468	I	I	V	I	I	I	I	I	I	I	I	I	I	I	I	I
Asn 232	Glu 179	N	N	N	N	N	N	N	N	N	N	N	N	N	N	N	N
-	Tyr 467	-	-	-	-	-	-	-	-	-	-	-	-	-	-	-	-
-	Ser 183	-	-	-	-	-	-	-	-	-	-	-	-	-	-	-	-
Lys 300	Lys 305	K	K	K	K	K	K	K	K	K	K	K	K	K	K	K	K

ZmPAO1	MmAPAO	I1L5L5	K4AYE9	K7LI87	K7ME99	L7VGD5	M1A995	M5X3Z6	Q4H439	Q9FNA2	R0FM29	S8EIZ2	U5G093	V4LC82	V4TFW4	V7BV40
Tyr 437	Thr 469	Y	Y	Y	Y	Y	Y	Y	Y	Y	Y	Y	Y	Y	Y	Y
Phe 401	-	Y	Y	Y	Y	Y	Y	Y	Y	Y	Y	Y	Y	Y	Y	Y
-	Cys 181	-	-	-	-	-	-	-	-	-	-	-	-	-	-	-
-	Asn 308	-	-	-	-	-	-	-	-	-	-	-	-	-	-	-
Glu 168	Val 182	E	E	E	E	E	E	E	E	E	E	E	E	E	E	E
Tyr 296	Tyr 425	Y	Y	Y	Y	Y	Y	Y	Y	Y	Y	Y	Y	Y	Y	Y
Glu 60	His 59	V	A	A	A	A	A	V	A	A	A	A	A	A	A	A
Tyr 167	Tyr 199	F	F	F	F	L	F	F	F	F	F	F	F	F	F	F
Tyr 163	Ser 468	I	I	I	I	I	F	I	I	I	I	I	I	I	I	I
Asn 232	Glu 179	N	N	N	N	N	N	N	N	N	N	N	N	N	N	N
-	Tyr 467	-	-	-	-	-	-	-	-	-	-	-	-	-	-	-
-	Ser 183	-	-	-	-	-	-	-	-	-	-	-	-	-	-	-
Lys 300	Lys 305	K	K	K	K	K	K	K	K	K	K	K	K	K	K	K

Subfamily 2

ZmPAO1	MmAPAO	A0A0D3H627	A0A0D3HCW1	A0A0D9XIZ7	A0A0D9XIZ8	A0A0D9XJ20	A0A0E0M0H0	A0A0E0M0H1	A0A0E0M0H2	A0A0E0MLW4	B8BGH2	D7TDE4	D7TDE5	D7TDE8	F2DTW7	I1I2U3	I1I3T4
Tyr 437	Thr 469	Y	Y	Y	Y	Y	Y	Y	Y	Y	Y	Y	Y	Y	Y	Y	Y
Phe 401	-	F	F	F	F	F	F	F	F	F	F	F	Y	Y	F	Y	F
-	Cys 181	-	-	-	-	-	-	-	-	-	-	-	-	-	-	-	-
-	Asn 308	-	-	-	-	-	-	-	-	-	-	-	-	-	-	-	-
Glu 168	Val 182	E	E	E	E	E	E	E	E	E	E	E	E	E	E	E	E
Tyr 296	Tyr 425	Y	Y	Y	F	Y	Y	Y	Y	Y	Y	Y	Y	Y	Y	Y	Y
Glu 60	His 59	E	E	E	E	E	E	E	E	E	E	E	E	S	E	E	E
Tyr 167	Tyr 199	Y	Y	Y	Y	Y	Y	Y	Y	Y	Y	Y	Y	F	F	Y	F
Tyr 163	Ser 468	F	C	Y	Y	Y	F	F	F	Y	C	Y	I	Y	Y	F	Y
Asn 232	Glu 179	N	N	N	N	A	N	N	N	N	N	N	N	K	N	N	N
-	Tyr 467	-	-	-	-	-	-	-	-	-	-	-	-	-	-	-	-
-	Ser 183	-	-	-	-	-	-	-	-	-	-	-	-	-	-	-	-
Lys 300	Lys 305	K	K	K	P	K	K	K	K	K	K	K	K	K	K	K	K

ZmPAO1	MmAPAO	J3LKX2	J3LKX3	K4A8M0	K4A8M6	K4A8T5	K4A8X1	K4A901	K7TH00	M0VYV1	M0XC59	M7YHU7	M7ZHC5	O64411	Q0J291	Q6H5M8	Q93WC0
Tyr 437	Thr 469	Y	Y	Y	Y	Y	Y	Y	Y	Y	Y	Y	Y	Y	Y	Y	Y
Phe 401	-	F	F	Y	Y	Y	Y	F	F	Y	F	F	F	F	F	F	Y
-	Cys 181	-	-	-	-	-	-	-	-	-	-	-	-	-	-	-	-
-	Asn 308	-	-	-	-	-	-	-	-	-	-	-	-	-	-	-	-
Glu 168	Val 182	E	A	E	E	E	E	E	E	E	E	E	-	E	E	E	E
Tyr 296	Tyr 425	Y	Y	Y	Y	Y	Y	Y	Y	Y	Y	Y	Y	Y	Y	Y	Y
Glu 60	His 59	E	E	E	E	E	E	E	E	E	E	E	E	E	E	E	E
Tyr 167	Tyr 199	Y	Y	Y	Y	Y	Y	Y	Y	Y	F	Y	-	Y	Y	Y	Y
Tyr 163	Ser 468	F	F	Y	Y	Y	Y	Y	Y	Y	Y	F	-	Y	F	F	Y
Asn 232	Glu 179	N	N	N	N	N	N	N	N	N	N	N	N	N	N	N	N
-	Tyr 467	-	-	-	-	-	-	-	-	-	-	-	-	-	-	-	-
-	Ser 183	-	-	-	-	-	-	-	-	-	-	-	-	-	-	-	-
Lys 300	Lys 305	K	K	K	K	K	K	K	K	K	K	K	K	K	K	K	K

Table S4: Continued

Subfamily 3

ZmPAO1	MmAPAO	A0A022QPJ5	A0A059CNL9	A0A061GKP9	A0A067F3K9	A0A067KFR9	A0A078EXI3	A0A078GXG7	A0A078I0C3	A0A078IW94	A0A087GI24	A0A0A0LFR4	A0A0B0N8V3	A0A0D2PT72	A0A0D2RT44	A2Q567	B9RK67
Tyr 437	Thr 469	T	T	T	T	T	T	T	T	T	T	T	T	T	T	T	T
Phe 401	-	-	-	-	-	-	-	-	-	-	-	-	-	-	-	-	-
-	Cys 181	T	T	T	T	T	T	T	T	T	T	T	T	T	T	T	T
-	Asn 308	V	V	V	V	V	V	V	V	V	V	V	V	V	V	V	V
Glu 168	Val 182	F	Y	Y	Y	Y	Y	Y	Y	Y	Y	Y	Y	Y	Y	Y	Y
Tyr 296	Tyr 425	Y	Y	Y	Y	Y	Y	Y	Y	Y	Y	Y	Y	Y	Y	Y	Y
Glu 60	His 59	H	H	H	H	H	H	H	H	H	H	H	H	H	H	H	H
Tyr 167	Tyr 199	Y	Y	Y	Y	Y	Y	Y	Y	Y	Y	Y	Y	Y	Y	Y	Y
Tyr 163	Ser 468	S	S	S	S	S	S	S	S	S	S	S	S	S	S	S	S
Asn 232	Glu 179	Q	Q	Q	Q	Q	Q	Q	Q	Q	Q	Q	Q	Q	Q	E	Q
-	Tyr 467	Y	Y	Y	Y	Y	Y	Y	Y	Y	Y	Y	Y	Y	Y	Y	Y
-	Ser 183	T	T	T	T	T	T	T	T	T	T	T	T	T	T	T	T
Lys 300	Lys 305	K	K	K	K	K	K	K	K	K	K	K	K	K	K	K	K

ZmPAO1	MmAPAO	F6HGW1	I1L1H1	I1MSH2	K4BZE2	K4CDQ7	K7MBX1	M1BIG2	M1BXE0	M1DUC0	M5VPD8	Q84XG2	Q9SU79	R0F4R6	V4MCU1	V4UKF2	V7B1I2
Tyr 437	Thr 469	T	T	T	T	T	T	T	T	T	T	T	T	T	T	T	T
Phe 401	-	-	-	-	-	-	-	-	-	-	-	-	-	-	-	-	-
-	Cys 181	T	T	T	T	T	T	T	T	H	T	T	T	T	T	T	T
-	Asn 308	V	V	V	V	V	V	V	V	V	V	V	V	V	V	V	V
Glu 168	Val 182	Y	Y	Y	Y	Y	Y	Y	Y	Y	Y	Y	Y	Y	Y	Y	Y
Tyr 296	Tyr 425	Y	Y	Y	Y	Y	Y	Y	Y	Y	Y	Y	Y	Y	Y	Y	Y
Glu 60	His 59	H	H	H	H	H	H	H	H	H	H	H	H	H	H	H	H
Tyr 167	Tyr 199	Y	Y	Y	Y	Y	Y	Y	Y	Y	Y	Y	Y	Y	Y	Y	Y
Tyr 163	Ser 468	S	S	S	S	S	S	S	S	S	S	S	S	S	S	S	S
Asn 232	Glu 179	Q	Q	Q	Q	Q	Q	Q	Q	N	Q	Q	Q	Q	Q	Q	Q
-	Tyr 467	Y	Y	Y	Y	Y	Y	Y	Y	Y	Y	Y	Y	Y	Y	Y	Y
-	Ser 183	T	T	T	T	T	T	T	T	S	T	T	T	T	T	T	T
Lys 300	Lys 305	K	K	K	K	K	K	K	K	K	K	K	K	K	K	K	K

ZmPAO1	MmAPAO	V7CBE3	W9QJP6
Tyr 437	Thr 469	T	T
Phe 401	-	-	-
-	Cys 181	T	T
-	Asn 308	V	V
Glu 168	Val 182	C	Y
Tyr 296	Tyr 425	Y	Y
Glu 60	His 59	H	H
Tyr 167	Tyr 199	Y	Y
Tyr 163	Ser 468	S	S
Asn 232	Glu 179	Q	Q
-	Tyr 467	Y	Y
-	Ser 183	T	T
Lys 300	Lys 305	K	K

Subfamily 4

ZmPAO1	MmAPAO	A0A0A0KYS9	A0A0D2RCX0	A0A0D2T3J4	A0A0D3G1I1	A0A0D3G1I2	A0A0D9WAZ4	A0A0D9ZS90	A0A0E0KVF6	A0A0E0PFI2	A0A1D6DYV6	A0A022R2Z4	A0A022RSV7	A0A059ADP6	A0A059AEF1	A0A059DGU2	A0A061DK84
Tyr 437	Thr 469	S	S	S	S	S	S	S	T	S	T	S	S	S	S	S	S
Phe 401	-	-	-	-	-	-	-	-	-	-	-	-	-	-	-	-	-
-	Cys 181	R	R	R	R	R	R	R	R	R	R	R	R	R	R	R	R
-	Asn 308	E	E	E	E	E	E	E	E	E	E	E	E	E	E	E	E
Glu 168	Val 182	M	M	M	L	L	L	L	M	L	M	M	M	M	M	M	M
Tyr 296	Tyr 425	Y	Y	Y	Y	Y	Y	Y	Y	Y	Y	Y	Y	Y	Y	Y	Y
Glu 60	His 59	H	H	H	H	H	H	H	H	H	H	H	H	H	H	H	H
Tyr 167	Tyr 199	E	E	A	E	E	E	E	E	E	E	E	E	V	E	E	A
Tyr 163	Ser 468	G	G	G	G	G	G	G	G	G	G	G	G	G	G	G	G
Asn 232	Glu 179	-	-	-	-	-	-	-	-	-	-	-	-	-	-	-	-
-	Tyr 467	P	P	P	S	S	S	S	T	S	T	Q	P	Q	Q	P	P
-	Ser 183	E	G	G	W	W	W	W	E	W	E	W	W	W	W	E	W
Lys 300	Lys 305	K	K	K	K	K	K	K	K	K	K	K	K	K	K	K	K

Table S4: Continued

A0A061DLV8	A0A067EQD2	A0A067KL71	A0A067KNR0	A0A072UW35	A0A078D8A2	A0A078EAX4	A0A078I134	A0A078IRH4	A0A087GZ44	A0A087H4U1	A2XYT9	B4F9F6	B6SV76	B6SYR8	B8AUI2
S	S	S	S	S	S	S	S	S	S	S	S	S	S	T	T
-	-	-	-	-	-	-	-	-	-	-	-	-	-	-	-
R	R	R	R	R	R	R	R	R	R	R	R	R	R	R	R
E	E	E	E	E	M	E	M	E	E	E	E	E	E	E	E
M	M	M	M	M	M	M	M	M	M	M	L	L	L	M	M
Y	Y	Y	Y	Y	Y	Y	Y	Y	Y	Y	Y	Y	Y	Y	Y
H	H	H	H	H	H	H	H	H	H	H	H	H	H	H	H
A	E	E	E	E	E	E	E	E	E	E	E	E	E	E	E
G	G	G	G	G	G	G	G	G	G	G	G	G	G	G	G
-	-	-	-	-	-	-	-	-	-	-	-	-	-	-	-
P	Q	Q	P	P	A	P	A	P	P	P	S	S	S	T	T
W	W	W	W	E	W	E	W	W	E	G	W	W	E	E	E
K	K	K	K	K	K	K	K	K	K	K	K	K	K	K	K

B9H3J5	B9H864	B9S6G9	B9SJL5	C5YA47	C5YG61	D7TDQ5	E0CTZ8	F2DFX4	G7J7X8	I1J1Z5	I1J380	I1JJ00	I1KXW2	I1LM64	I1MBQ3
S	S	S	S	S	T	S	S	T	S	T	A	S	S	S	S
-	-	-	-	-	-	-	-	-	-	-	-	-	-	-	-
R	R	R	R	R	R	R	R	R	R	R	R	R	R	R	R
E	E	E	E	E	E	E	E	E	E	E	E	E	E	E	E
M	M	M	M	L	M	M	M	M	M	M	L	M	L	M	M
Y	Y	Y	Y	Y	Y	Y	Y	Y	Y	Y	Y	Y	Y	Y	Y
H	H	H	H	H	H	H	H	H	H	H	H	H	H	H	H
E	E	E	A	E	E	E	E	E	E	E	E	E	E	E	E
G	G	G	G	G	G	G	G	G	G	G	G	G	G	G	G
-	-	-	-	-	-	-	-	-	-	-	-	-	-	-	-
Q	P	P	Q	S	T	Q	P	T	Q	T	S	T	P	Q	Q
W	E	W	W	W	E	W	W	E	W	E	W	E	E	W	A
K	K	K	K	K	K	K	K	K	K	K	K	K	K	K	K

I1MZJ1	I1PQ13	I1PQS6	I1PQS7	J3M1J7	J3M2I5	J3M2I6	K3Y6Y2	K3Z5Q7	K3Z5R8	K4BA09	K4BFF9	K4CEL6	K4DBH4	K7KAF6	K7TNR6
S	T	S	S	T	S	S	T	S	S	S	S	S	S	Y	T
-	-	-	-	-	-	-	-	-	-	-	-	-	-	-	-
R	R	R	R	R	R	R	R	R	R	R	R	R	R	R	R
E	E	E	E	E	E	E	E	E	E	E	E	E	E	E	E
M	M	L	L	M	L	L	M	L	L	M	M	M	M	M	M
Y	Y	Y	Y	Y	Y	Y	Y	Y	Y	Y	Y	Y	Y	Y	Y
H	H	H	H	H	H	H	H	H	H	H	H	H	H	H	H
E	E	E	E	E	E	E	E	E	E	E	E	E	E	E	E
G	G	G	G	G	G	G	G	G	G	G	G	G	G	G	G
-	-	-	-	-	-	-	-	-	-	-	-	-	-	-	-
Q	T	S	S	T	S	S	T	S	S	Q	Q	P	P	Q	T
W	E	W	W	E	W	W	E	W	W	W	E	W	W	W	E
K	K	K	K	K	K	K	K	K	K	K	K	K	K	K	K

M0RTD5	M0U284	M0VUD5	M1BAZ8	M1BQU1	M1BQU3	M1C867	M1CT70	M7Z7G8	Q7X809	Q7XR46	Q9LYT1	Q9SKX5	R0FMX3	S8E4F7	U5FLG7
T	S	A	S	S	S	S	S	S	T	S	S	S	S	S	S
-	-	-	-	-	-	-	-	-	-	-	-	-	-	-	-
R	R	R	R	R	R	R	R	R	R	R	R	R	R	R	R
E	E	E	E	E	E	E	E	E	E	E	E	E	E	E	E
M	L	L	M	M	M	M	M	L	M	L	M	M	M	M	M
Y	Y	Y	Y	Y	Y	Y	Y	Y	Y	Y	Y	Y	Y	Y	Y
H	H	H	H	H	H	H	H	H	H	H	H	H	H	H	H
E	E	E	E	A	E	E	E	E	E	E	E	E	E	E	Q
G	G	G	G	G	G	G	G	G	G	G	G	G	G	G	G
-	-	-	-	-	-	-	-	-	-	-	-	-	-	-	-
T	S	S	P	Q	Q	Q	P	S	T	S	P	P	P	T	Q
E	W	W	W	W	W	E	W	W	E	W	E	E	W	W	W
K	K	K	K	K	K	K	K	K	K	K	K	K	K	K	K

Table S4: Continued

V4KBX8	V4LNX9	V4LR45	V4M9N7	V4MK60	V4SS93	V4TFE5	V4VD50	V7B817	V7BLV1	V7BNY1	W5AQH0	W5BA09	W9RTP2
S	S	S	S	S	S	S	S	S	S	S	A	A	S
-	-	-	-	-	-	-	-	-	-	-	-	-	-
R	R	R	R	R	R	R	R	R	R	R	R	R	R
E	E	E	E	E	E	E	E	E	E	E	E	E	E
M	M	M	M	M	M	M	M	M	M	M	L	L	M
Y	Y	Y	Y	Y	Y	Y	Y	Y	Y	Y	Y	Y	Y
H	H	H	H	H	H	H	H	H	H	H	H	H	H
D	E	E	E	E	E	E	E	E	E	E	E	E	-
G	G	G	G	G	G	G	G	G	G	G	G	G	G
-	-	-	-	-	-	-	-	-	-	-	-	-	-
Q	P	P	P	P	Q	P	P	Q	Q	P	S	S	P
E	E	E	E	E	W	W	W	W	W	G	W	W	E
K	K	K	K	K	K	K	K	K	K	K	K	K	K

Subfamily 5

ZmPAO1	MmAPAO	A0A0D9V4T9	A0A0D9YDX7	A0A0E0JN44	A2WUB8	C0PE40	C5XI79	Q5NAI7
Tyr 437	Thr 469	T	T	T	T	T	T	T
Phe 401	-	-	-	-	-	-	-	-
-	Cys 181	T	T	T	T	A	T	T
-	Asn 308	V	V	V	V	V	V	V
Glu 168	Val 182	D	D	D	D	D	D	D
Tyr 296	Tyr 425	Y	Y	Y	Y	Y	Y	Y
Glu 60	His 59	Q	Q	Q	Q	Q	Q	Q
Tyr 167	Tyr 199	Y	Y	Y	Y	Y	Y	Y
Tyr 163	Ser 468	S	S	S	S	S	S	S
Asn 232	Glu 179	E	E	E	E	E	E	E
-	Tyr 467	Y	Y	Y	Y	Y	Y	Y
-	Ser 183	T	T	T	T	T	T	T
Lys 300	Lys 305	K	K	K	K	K	K	K

Table S3: Plant PAOs active site analysis. The aminoacidic profile for each position of the active site was retrieved for each sequence within each subfamily from the Multiseq module of the VMD software.

Table S5

A

	ZmPAO1		FMS1		MmAPAO	
	GMQE	QMEAN	GMQE	QMEAN	GMQE	QMEAN
Cs4g14150.1_CsPAO4	0.52	-5.98	0.48	-6.89	0,58	-4,39

B

ZmPAO1	MmAPAO	CsPAO4
Tyr 437	Thr 469	T
Phe 401	-	-
-	Cys 181	T
-	Asn 308	V
Glu 168	Val 182	Y
Tyr 296	Tyr 425	Y
Glu 60	His 59	H
Tyr 167	Tyr 199	Y
Tyr 163	Ser 468	S
Asn 232	Glu 179	Q
-	Tyr 467	Y
-	Ser 183	T
Lys 300	Lys 305	K

Table S5: CsPAO4 model quality and active site analysis. A- Model quality parameters. GMQE and QMEAN of models obtained with three templates. ZmPAO1 (pdb code 3KU9), FMS1 (pdb code 1XPQ) and MmAPAO (pdb code 5MBX). B- CsPAO4 active site analysis. The aminoacidic profile for each positio of the active site was retrieved for each sequence within each subfamily from the Multiseq module of the VMD software.
